# Causal machine learning reveals heterogeneous effects of loss versus gain framing on retirement savings

**DOI:** 10.1038/s41598-026-76813-1

**Published:** 2026-11-02

**Authors:** Kevin Bösch, Oliver Müller, Markus Weinmann, Stefan Feuerriegel

**Affiliations:** 1 Paderborn University, Paderborn, Germany; 2 University of Cologne, Cologne, Germany; 3 LMU Munich, Munich, Germany; 4 Munich Center for Machine Learning, Munich, Germany

**Keywords:** Mathematics and computing, Psychology, Psychology

## Abstract

Abstract: As Western countries face rapidly aging populations, insufficient retirement savings threaten individual financial security and the sustainability of social welfare systems. Behavioral interventions such as loss–gain message framing are often proposed as scalable solutions, yet evidence on their effectiveness is mixed, largely because little is known about when and for whom they work. We analyze heterogeneity in the effects of loss–gain framing on real-life, high-stakes retirement saving decisions. In a large-scale randomized field experiment ($N = 22{,}919$) with a European retirement savings company, individuals were randomized to loss- or gain-framed reminders designed to increase participation (extensive margin) and savings amount (intensive margin). Using causal machine learning, we estimate heterogeneous treatment effects across socioeconomic characteristics and validate them in a second, independent experiment ($N = 18{,}288$). In this validation experiment, for the 25% most responsive to loss framing, conversion is estimated to rise by 9.4 percentage points relative to gain framing, whereas for the 25% most responsive to gain framing it is expected to fall by 8.4 points. For savings amount, we estimate that loss framing raises contributions for 64% of contributors (by €737 on average) but backfires for the rest (by €615 on average). Overall, a personalized targeting policy based on causal machine learning is expected to increase participation by 51% (from 4.53% to 6.86%) and average savings by 18% (from €2,062 to €2,442 per converted customer) relative to uniform loss framing.

## Introduction

As most Western countries face rapidly aging populations^1,2^, insufficient retirement savings pose serious challenges both for individuals and for the sustainability of social welfare systems. In the United States, for example, 39% of households are at risk of not being able to maintain their standard of living in retirement^3^, and current estimates suggest that public expenditure on retirement will increase from 8.8% of GDP to 10% in 2050 in the OECD-32 states^4^. In response to the rising costs of state pension provision, most OECD countries have introduced retirement schemes that shift responsibility for retirement provision onto individuals, including 401(k) plans in the United States, the Riester pension in Germany, or the “third pillar” in Switzerland^5^. However, despite a broad array of retirement savings programs, participation and contribution levels remain low and contribute to a widely documented retirement savings gap. Old-age poverty has harsh consequences, such as lower quality of life^6,7^, higher mortality^8–11^, lower life satisfaction^12^, and poorer mental health^13,14^. Closing this savings gap is among the central challenges of behavioral public policy.

To address the retirement savings crisis, behavioral interventions have been developed and tested to motivate people to increase their savings. One well-known example is the “Save More Tomorrow” program in the United States^15^, in which employees are automatically enrolled in savings plans and pre-commit to automatically increase their retirement saving rate whenever they receive a future pay raise^1^. The program has been rolled out to more than 15 million people, and average savings rates increased by roughly 10 percentage points over three years^1^. Such lightweight behavioral interventions are attractive because they can often be implemented at low cost, scaled to large populations, and embedded directly into existing financial systems. As a result, behavioral interventions to promote savings have been tested around the world^16–21^.

One commonly studied behavioral intervention is message framing^22^, which manipulates whether information is presented as a potential gain or a potential loss. This loss–gain framing is grounded in prospect theory, which states that people evaluate outcomes relative to a reference point and often perceive losses as more consequential than equivalent gains^23^. As Kahneman and Tversky^23^ put it, “losses loom larger than gains.” For instance, a retirement savings message could highlight the gain of “having an extra €500 available each month in retirement” or, alternatively, the loss of “having €500 less to spend each month in retirement.” Previously, loss–gain framing has been applied as a behavioral intervention across various domains such as medicine (e.g., promoting cancer screening^24^ or vaccination^25^), climate change^26^, factory productivity^27^, student loans^28^, and consumer decision-making (e.g., promoting the adoption of green energy^29^). However, the overall empirical findings are inconclusive. For example, in the context of health care, O’Keefe and Jensen^30^ found a small but statistically significant difference between frames in the context of breast cancer self-examination, but no effects for other types of disease-prevention actions, such as promoting vaccinations^25^.

In the retirement savings context, some studies found that loss frames outperform gain frames^31,32^, while others report the opposite^33,34^. These inconsistencies suggest that framing effects in retirement savings are context-dependent and may vary across individuals. Prior studies have shown that framing effects are moderated by gender^33,35,36^, wealth^33–35^, and financial literacy^33,34,37^. In particular, lower financial literacy is associated with stronger framing effects^33,34^, and less financial freedom is associated with increased framing effects^33–35^. Regarding gender, some studies^33^ find that men are generally more susceptible to framing than women, but women are more affected by loss framing than men^36,38^. Together, these studies show that framing effects are not uniform but depend on individual characteristics.

These mixed effects may stem from a key limitation of prior work: existing analyses focus predominantly on the average treatment effect (ATE), which captures whether a behavioral intervention is effective at the population level. However, averages mask important heterogeneity in behavior across individuals^39^. In the behavioral sciences, it is well established that nearly all behavioral phenomena occur under some conditions but not others^39^. Recent reviews further emphasize that heterogeneity is the rule rather than the exception and highlight the need to identify *moderators*, i.e., individual or contextual characteristics that determine *when* and *for whom* interventions are effective^40^. Moreover, focusing only on averages risks creating a false impression of ineffectiveness: positive effects for some individuals can be canceled out by negative effects for others, so that the underlying variation remains invisible^41^. By averaging across such subgroups, existing studies may underestimate effects and risk labeling interventions as ineffective when, in fact, they are effective for specific subpopulations.

Here, we hypothesize that the relative effectiveness of loss- versus gain-framed messages in promoting retirement savings varies across individual-level characteristics. Human behavior is inherently heterogeneous and shaped by factors such as age^42^, education^43^, type of work^44^, and income^45^. Accordingly, individuals with different socioeconomic characteristics may respond systematically differently to message frames, even when the underlying financial product is identical. Rather than testing whether a framing intervention is effective compared to no framing, we study which frame works best for whom. Hence, identifying who benefits from which intervention is central for designing behavioral policies that are both effective and acceptable^46^. To do so, we use causal machine learning (Causal ML)^47–49^ to estimate individual-level treatment effects of loss versus gain framing as a function of observable socioeconomic characteristics. Unlike conventional subgroup or interaction analyses, Causal ML learns treatment effect heterogeneity from data in a flexible, non-linear, data-driven manner without strong parametric assumptions^50^. This allows us to generate individual-level treatment effects (i.e., the conditional average treatment effect, CATE) rather than only describing average differences across a few broad subgroups of the population. The predictions can then inform targeting policies that personalize message framing and aim to maximize overall retirement savings contributions while avoiding negative effects for specific subgroups.

Understanding effect heterogeneity is relevant for both science and policy. First, it allows policy-makers and practitioners to move beyond one-size-fits-all strategies and instead tailor message-framing interventions to the subgroups most likely to benefit^39,40,51^, thereby improving effectiveness. In the case of retirement savings, this could mean offering gain-framed messages to some individuals while emphasizing loss-framed messages for others, leading to more personalized financial advice. Second, studying heterogeneity contributes to our theoretical understanding by formalizing the mechanisms through which framing operates in high-stakes economic decisions with long-term consequences, an area where psychological processes remain poorly understood. This aligns with calls to ground behavioral interventions more firmly in the cognitive and inferential processes that drive behavior^52^. Third, relying solely on average effects can mask that individuals react very differently to the same intervention: effects that appear null on average can conceal subpopulations with positive and negative responses that are not known *a priori* and are defined through complex, often non-linear characteristics. Identifying these subpopulations makes it possible to assign each individual the frame they respond to and thereby to generate higher overall participation and savings.

**Fig. 1:**
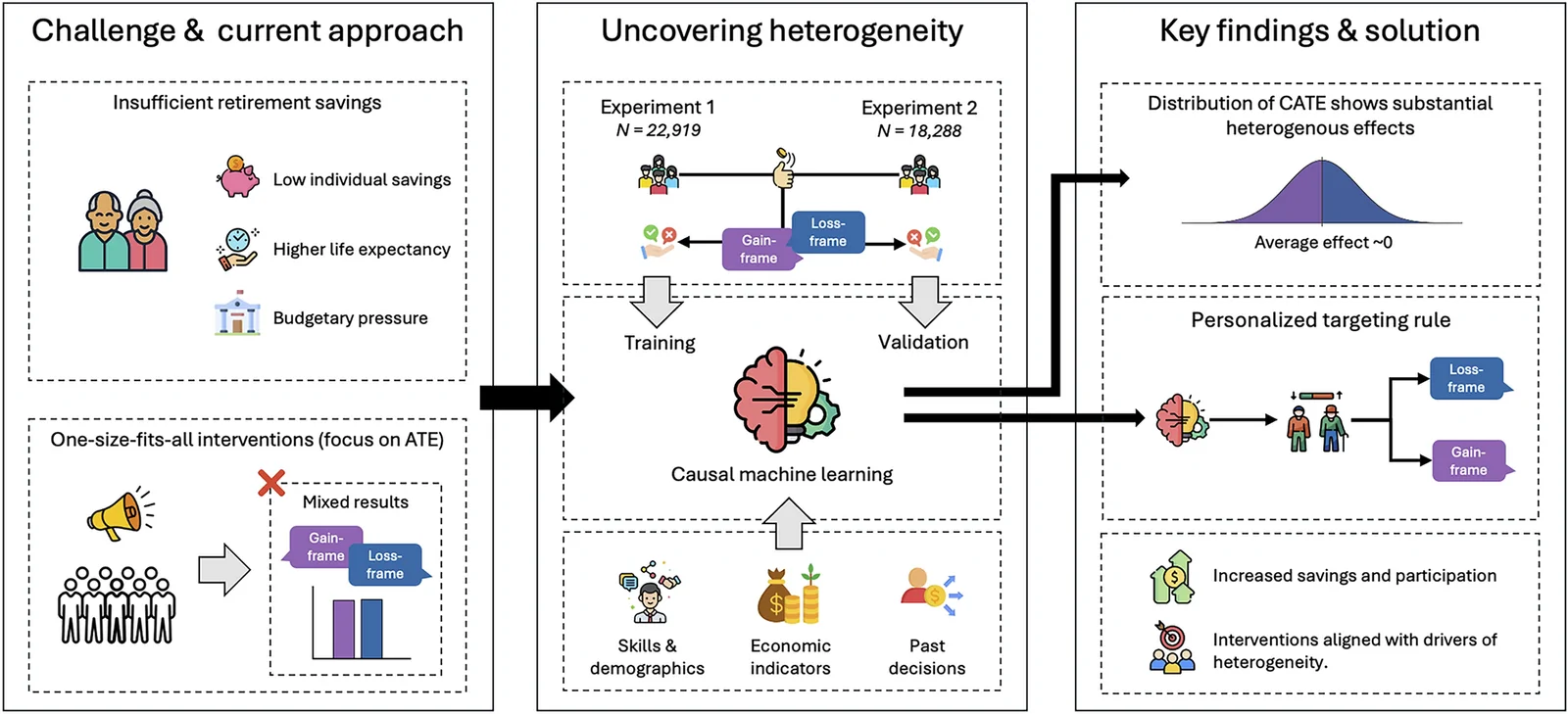
Overview of our research framework. **Left:** Our objective is to understand individual-level differences in *when* loss versus gain framing increases retirement savings. Prior research has largely relied on “one-size-fits-all” strategies, in which the same behavioral intervention is uniformly applied to the entire population based on the average treatment effect. However, focusing only on average treatment effects can mask individual differences and thus the fact that interventions are effective for some while backfiring for others. **Center:** To address this limitation, we conducted two large-scale randomized field experiments ($N = 22{,}919$ and $N = 18{,}288$). Using causal machine learning, we analyze how responsiveness to loss versus gain framing varies across individuals by estimating the heterogeneous treatment effects conditional on a rich set of covariates, including demographics, skills, and past saving behavior. **Right:** Our analysis reveals substantial heterogeneity in individual responses, showing that individuals with different characteristics can react in opposite ways to the same framing. Finally, we developed a personalized targeting rule that assigns the message frame with the highest expected effect to each individual, thereby demonstrating the benefits of exploiting heterogeneity in individual-level responses even though the population-average effects are close to zero.

In this paper, we analyze heterogeneity in the effectiveness of loss–gain framing on retirement savings (see Fig. 1). Specifically, we examine two outcome dimensions: (i) the *extensive margin*, capturing whether individuals choose to contribute at all, and (ii) the *intensive margin*, reflecting how much they contribute once they decide to save. Distinguishing between the extensive and intensive margins is important because focusing only on contribution volumes (i.e., intensive margin) risks favoring wealthier individuals, whereas, from a societal perspective, it may be more important to encourage broad participation (i.e., extensive margin) to involve those most dependent on retirement savings.

We conducted two consecutive large-scale randomized field experiments in collaboration with a European retirement savings company (the majority of customers are in Switzerland, with the remainder elsewhere in Europe; all financial figures are converted to euros using the Swiss Franc–Euro exchange rate of approximately 1:1 that prevailed during the study period). This allowed us to observe real-world, high-stakes retirement savings decisions (the average individual savings amount across both experiments was €1,868). The first experiment ($N = 22{,}919$) collected data to train a causal forest model that estimates heterogeneous treatment effects. Based on the CATE estimates, we developed a personalized targeting rule that maps subgroups of individuals to the framing strategy that benefits them most. We used the data collected from the second randomized experiment ($N = 18{,}288$) to validate both the CATE estimates and the personalized targeting rule. Here, the second experiment acts as an out-of-sample validation, allowing us to assess the robustness and generalizability of our findings.

At the population level, loss- and gain-framed messages have similar aggregated effects on both the decision to save (extensive margin) and the amounts saved (intensive margin). The ATE, however, can mask substantial heterogeneity and be misinterpreted as evidence of ineffectiveness. Contrary to this, our Causal ML-based CATE analysis reveals large systematic differences in how individuals respond to loss versus gain framing. Focusing on the extensive margin (i.e., decision to save), we estimate that for the top-25% of individuals predicted to be most responsive to loss framing, exposure to loss- rather than gain-framed messages increases the participation rate by 9.4 percentage points. Conversely, for the top-25% predicted to be most responsive to gain framing, participation is expected to decrease by 8.4 percentage points when exposed to loss-framed messages. For the intensive margin (i.e., how much to save), loss framing increases contributions for 64% of the individuals, with an average increase of €737, but backfires for the others (i.e., reducing contributions by €615 on average). Overall, leveraging heterogeneity through a CATE-based personalization policy is expected to increase participation by 51% (from 4.53% to 6.86%) and average savings amounts by 18% (from €2,062 to €2,442 per converted customer) relative to uniform loss message framing. Comparing the personalization policy against uniform gain frame messages, participation increases by 66% and savings increase by 23%. These expected benefits clearly demonstrate the potential of personalized interventions beyond policies based on average effects.

Our Causal ML analysis identifies important drivers of differential effectiveness across socioeconomic characteristics. For the extensive margin (decision to save), loss frames tend to work better for customers with high information processing skills, while gain frames tend to work better for older customers (50+ years) and customers who have made many additional savings in the past. For the intensive margin (savings amounts), loss frames are more effective for customers with high computer skills, while gain frames are more effective for customers with small savings plans and for customers with blue-collar jobs (e.g., those with high handling, moving, construction skills). These patterns suggest that framing interventions operate through distinct mechanisms for extensive and intensive margins, which emphasizes the importance of tailoring behavioral interventions to the populations they are intended to reach. Beyond increasing overall effectiveness, such personalization matters because uniform interventions benefit some subpopulations while having unintended effects on others; leveraging this heterogeneity allows assigning each customer the frame they respond to. These results show how Causal ML can be used to design retirement communication policies that are behaviorally effective among populations of different characteristics.

### Results

We conducted two large-scale field experiments to study the effect of message framing on retirement savings. In collaboration with a European FinTech that offers so-called unit-linked retirement savings products (i.e., a life-insurance contract in which a proportion serves the life-insurance premium and a part is invested in funds), we experimentally manipulated the presentation of annual reminder messages sent to customers about their remaining tax-deductible savings amounts to encourage additional contributions. In total, 41,207 customers were randomly assigned to different message framings (i.e., $N = 22{,}919$ in Field Experiment 1 that took place in 2021 and $N = 18{,}288$ in Field Experiment 2 that took place in 2022). Across both experiments, a total of $N=1{,}986$ — $N=1{,}195$ in Experiment 1 and *N=791* in Experiment 2 — customers (4.82% across both experiments) made a contribution with an average saving amount of €1,868 to their retirement savings plan in response to the reminder messages.

Our empirical strategy is as follows (the full details are reported in Methods). In both experiments, customers were randomized individually within the partner company’s production system using a pseudo-random number generator, with assignment independent of covariates and prior behavior (a 2:1 loss-to-gain allocation in Field Experiment 1 and a 50:50 allocation in Field Experiment 2; covariate balance is reported separately by experiment in Tables S3 and S4 of the Supplementary Materials). Because treatment was assigned at random, the conditional average treatment effect (CATE) is identified as the difference in conditional outcome expectations under loss versus gain framing, given a vector of pre-treatment covariates. All covariates, that is, demographics, contract characteristics, prior saving behavior, and ESCO-based job skill profiles (Table S2), were measured before the intervention, and the gain-framed condition serves as the control group throughout. We estimate the CATE using a causal forest^47,48^, a nonparametric Causal ML estimator that models treatment effect heterogeneity without imposing parametric assumptions on how covariates modify the effect. The model is trained on data from Field Experiment 1, whereas all reported estimates (ATE, CATE, RATE) and all policy evaluations are computed on the held-out data from Field Experiment 2. Hence, the reported quantities are out-of-sample and are evaluated under a treatment assignment mechanism that is exogenous to the model^53^.

**Fig. 2:**
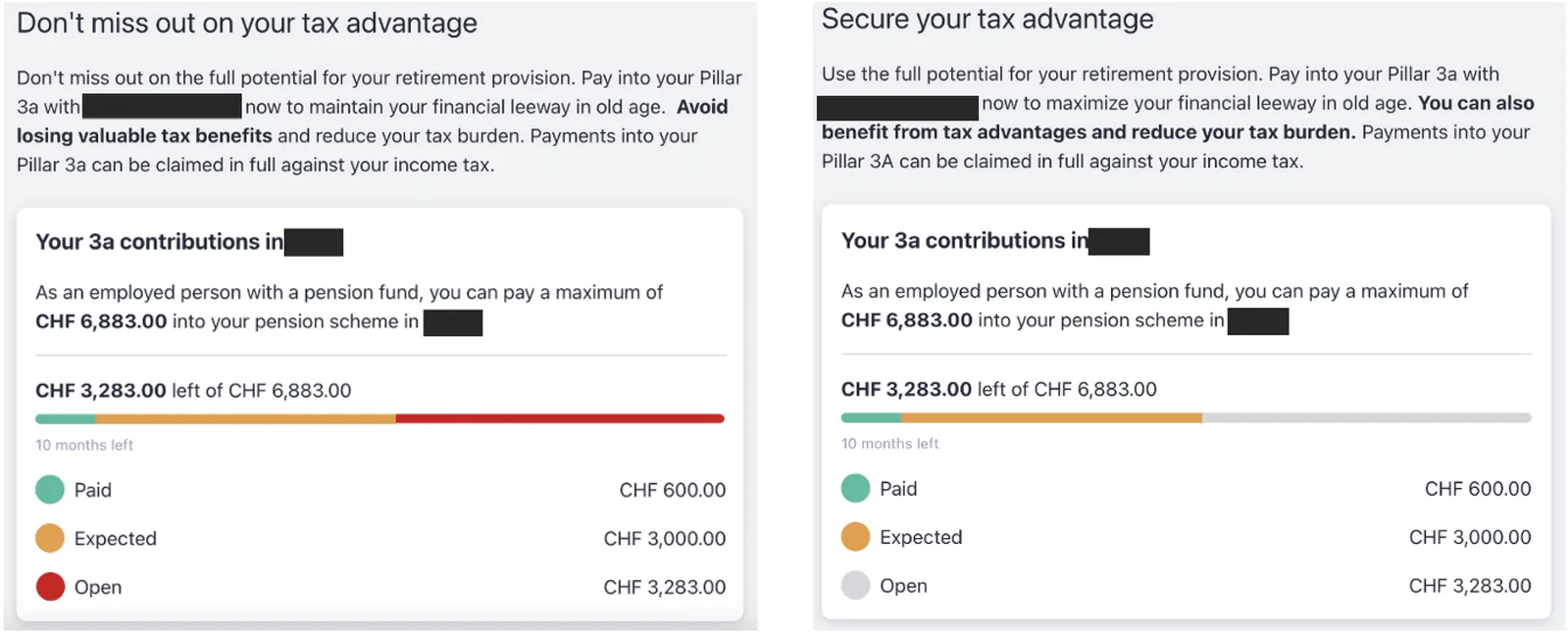
Screenshots with experimental conditions showing loss versus gain framing. Screenshot of the retirement savings app with loss framing (left) and gain framing (right) as deployed in this study. The treatment included textual cues (*“miss out,”* *“avoid losing”* vs. *“secure,”* *“benefit from”*) and color coding (red vs. silver-gray). In the illustrated example, the customer has already saved a total of €600 through recurring and/or one-time payments. It is further projected that the customer will save an additional €3,000 through recurring payments in the remaining months of the current calendar year. Therefore, €3,283 remain open to cover the full €6,883.00 tax-deductible amount in the current year.

Customers were randomly assigned to receive either loss- or gain-framed reminders. Loss-framed messages stressed the costs of not contributing to their retirement savings plan, while gain-framed messages emphasized the benefits of additional contributions. The interventions combined textual cues (e.g., “avoid losing valuable tax benefits” for loss framing versus “benefit from tax advantages” for gain framing) with color cues (a red bar chart for loss framing versus a silver-gray bar chart for gain framing). All other message content—including the amounts already contributed, expected contributions, and remaining deductible amounts—remained identical across conditions. Thus, any behavioral differences between the conditions reflect the causal effect of loss versus gain framing. Exemplary screenshots of the message in the FinTech’s wealth management app are shown in Fig. 2.

We evaluated how loss versus gain framing influenced retirement savings along two economic dimensions: (i) extensive margin (decision to save) and (ii) intensive margin (savings amount). The extensive margin captured whether individuals made an additional contribution, measured as a binary variable (i.e., converted: yes/no). The intensive margin captured the amount contributed by those who converted and was measured as a continuous savings amount (in €). These two dimensions therefore distinguish the decisions to save at all (extensive margin) from the decision of how much to contribute (intensive margin).

To analyze potential heterogeneity in the effect of loss- versus gain-framed messages on two outcomes, we trained a causal forest algorithm^47,48^ using a broad set of covariates with individual-level socioeconomic attributes and behaviors from each customer (e.g., customer age, contract valuation, historical payments; see the complete list in Supplementary Table S2). The causal forest estimates the individual-level response to the treatment via the CATE. For the extensive margin, the CATE quantifies, for each customer, the expected change in the probability of making an additional contribution when receiving a loss-framed rather than a gain-framed message (measured in percentage points). For the intensive margin, the CATE represents the expected change in the savings amount (in €) among those who decide to contribute. To evaluate the robustness and out-of-sample generalization of the causal forest, we used data from Field Experiment 1 to train the causal forest and data from Field Experiment 2 for a hold-out validation of the performance. Validation using independent randomized data is particularly valuable for identifying subgroup effects^54–56^, as it allows us to assess how the estimated personalization policy would perform under a treatment assignment mechanism that is exogenous to the model^53^.

### Heterogeneous effects on the extensive margin (decision to save)

#### Heterogeneity analysis

**Fig. 3:**
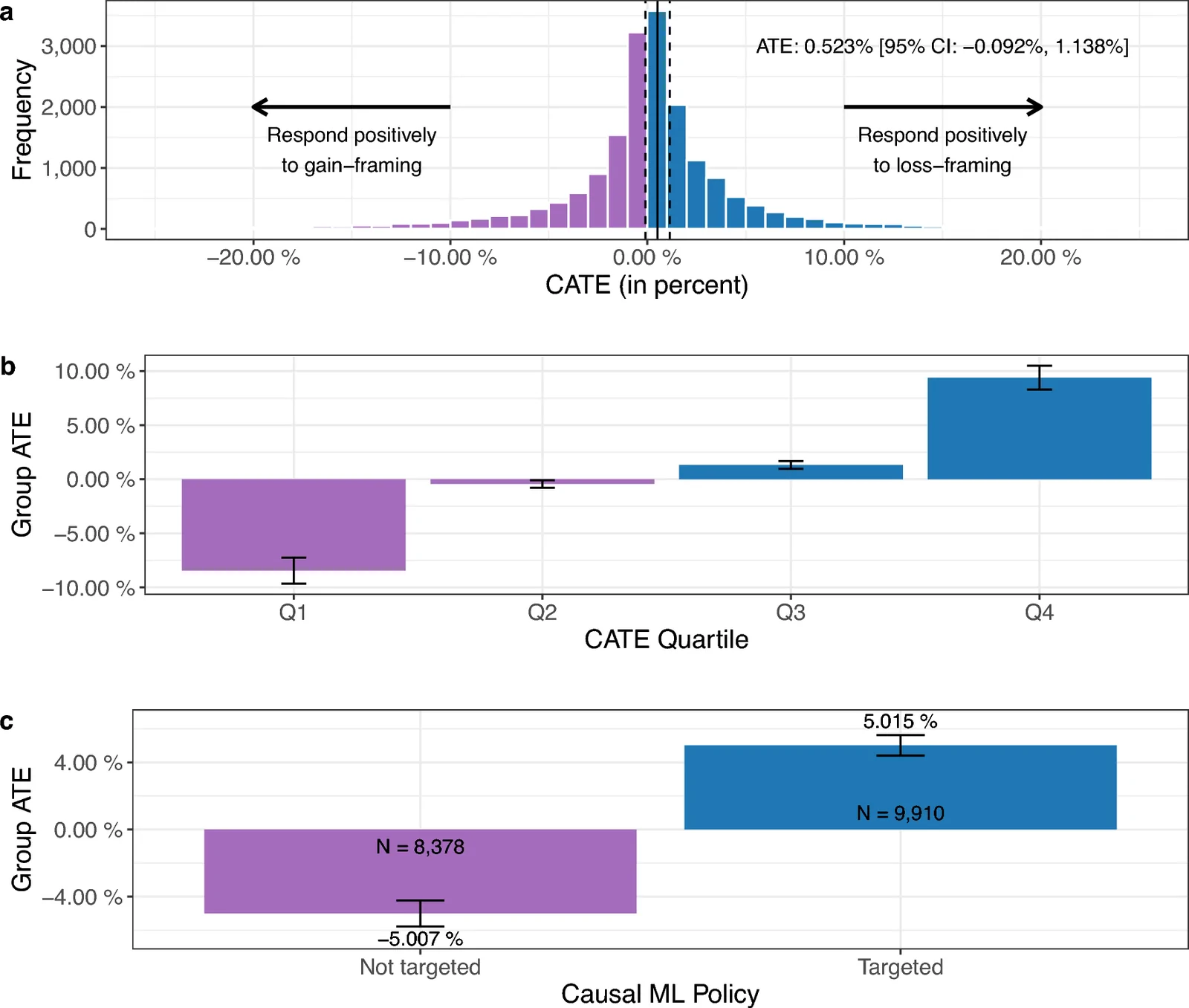
Heterogeneous effects of loss versus gain framing on conversion probability (extensive margin). This figure shows the estimated heterogeneity in the effect of message framing on customers’ probability of making an additional retirement savings contribution. The results are based on a causal forest^47,48^, which was trained on data from Field Experiment 1 and evaluated out-of-sample using data from Field Experiment 2. (**a**) Distribution of conditional average treatment effects (CATE) of sending a loss-framed message (versus a gain-framed message) on conversion probability, measured in percentage points. Positive CATE values indicate individuals who are more likely to convert under loss framing, whereas negative values indicate individuals for whom loss framing reduces conversion probability relative to gain framing. For comparison, we show the ATE (solid vertical line) with 95% CIs (dashed vertical line) estimated using a doubly robust augmented inverse propensity weighting (AIPW) method^57,58^. The X-axis is truncated at +/- 25.00% for better readability. (**b**) Average treatment effects (ATE) of sending a loss-framed message on conversion probability, estimated separately for quartiles of the predicted CATE distribution. The monotonic increase in ATEs across quartiles indicates that the model successfully ranks individuals by their responsiveness to loss versus gain framing. (**c**) Here, a Causal ML–based targeting policy is evaluated that assigns loss-framed messages to individuals with positive predicted CATEs (i.e., are targeted by the model) and gain-framed messages to individuals with negative predicted CATEs (i.e., are not targeted by the model). The uncertainty margins depicted in the plots represent 95% confidence intervals.

The CATE distribution reveals substantial heterogeneity in how individuals respond to loss- versus gain-framed messages (Fig. 3a). Here, positive CATE values identify individuals for whom the conversion probability increases when receiving a loss-framed message rather than a gain-framed message. Conversely, negative CATE values identify individuals who instead respond more positively to gain-framed messages. Overall, the CATE estimates range from −40 to +48 percentage points in conversion probability (first to third quartile: −1 to +2). Approximately 54% of the customers have a positive CATE, indicating that loss framing increases their likelihood of saving, while 46% have a negative CATE and thus respond more positively to gain framing as opposed to loss framing.

To formally test for effect heterogeneity, we inspected the targeting operator characteristic (TOC) (see Figure S3) and further computed the rank-weighted average treatment effect (RATE)^59^, including the corresponding 95% confidence interval (CI). The RATE estimate is 2.59 (95% CI: 1.72, 3.46) and thus statistically significant from zero. Together, this provides further evidence that heterogeneous effects of loss–gain framing are present.

Importantly, the pronounced heterogeneity is not apparent when examining the ATE. At the population level, the ATE of loss versus gain framing is +0.523 (95% CI:−0.092, 1.138) percentage points and thus close to zero, which could be interpreted as evidence that the type of frame does not matter. However, the ATE as a population-level estimate masks the fact that loss framing substantially increases conversion probabilities for a large subset of customers, while simultaneously decreasing conversion for others, which motivates the analysis here to move beyond the ATE and understand individual-level heterogeneity for whom framing interventions are effective.

#### Robustness checks

To assess the robustness of our model estimates, we follow established practice in Causal ML^60–62^ to assess effect heterogeneity at the group level. Specifically, we sort customers from the hold-out test set into quartiles based on their predicted CATEs and then estimate the ATEs within each quartile using augmented inverse propensity weighting (AIPW)^57,58^. This approach allows us to evaluate whether the ranking implied by the CATE predictions aligns with observed treatment effects in unseen data. The results show a clear and monotonic pattern (Fig. 3b). Overall, the ATE increases monotonically from the lowest to the highest CATE quartile, providing strong evidence that the causal forest successfully captures meaningful heterogeneity in treatment effects. This monotonic relationship between predicted and realized effects indicates that the CATE estimates are well-calibrated and that the model reliably ranks individuals by how they respond to loss- versus gain-framed messages.

To further assess effect heterogeneity, we grouped individuals by the sign of their estimated CATEs and then compared how these groups respond to message framing (Fig. 3c). Such binary grouping corresponds to a natural decision rule faced by a practitioner: whether an individual is expected to benefit more from a loss-framed or a gain-framed message. As a result, we yield a group with positive predicted CATE values ($N = 9{,}910$) that exhibits a large and statistically significant ATE, and a group with negative predicted CATEs ($N = 8{,}378$) that exhibits a negative and statistically significant ATE. Concretely, the ATE of loss-framed messages for individuals whom the causal forest model identified as responsive to such loss-framed messages was 5.015 percentage points (95% CI: 4.403, 5.627), whereas the ATE for those identified as *not* responsive to loss-framed messages was −5.007 percentage points (95% CI: −5.785, −4.229). This evidence suggests that a simple CATE-based policy can be implemented as a target rule, which assigns loss-framed messages to individuals with positive predicted CATEs and gain-framed messages to those with negative predicted CATEs. Importantly, these effects are evaluated out-of-sample, because the causal forest was trained on data from Field Experiment 1 and then assessed on data from Field Experiment 2.

#### Drivers of heterogeneity

To identify drivers that explain the heterogeneous responses to loss- versus gain-framed messages, we apply techniques from Explainable AI (XAI) to inspect the trained causal forest model^63^. These techniques allow us to translate the rich, non-linear structure learned by the causal forest into interpretable insights by revealing which observable characteristics contribute most to differences in treatment effects and how these effects vary across the population. This is particularly important in our setting, where framing responses depend on complex interactions between prior saving behavior, socioeconomic characteristics, and occupational attributes that are difficult to capture with linear models. By making these non-linear and interaction effects transparent, XAI has advantages over simple univariate comparisons, which typically require strong *a priori* assumptions about where heterogeneity lies and how it operates^64^.

**Fig. 4:**
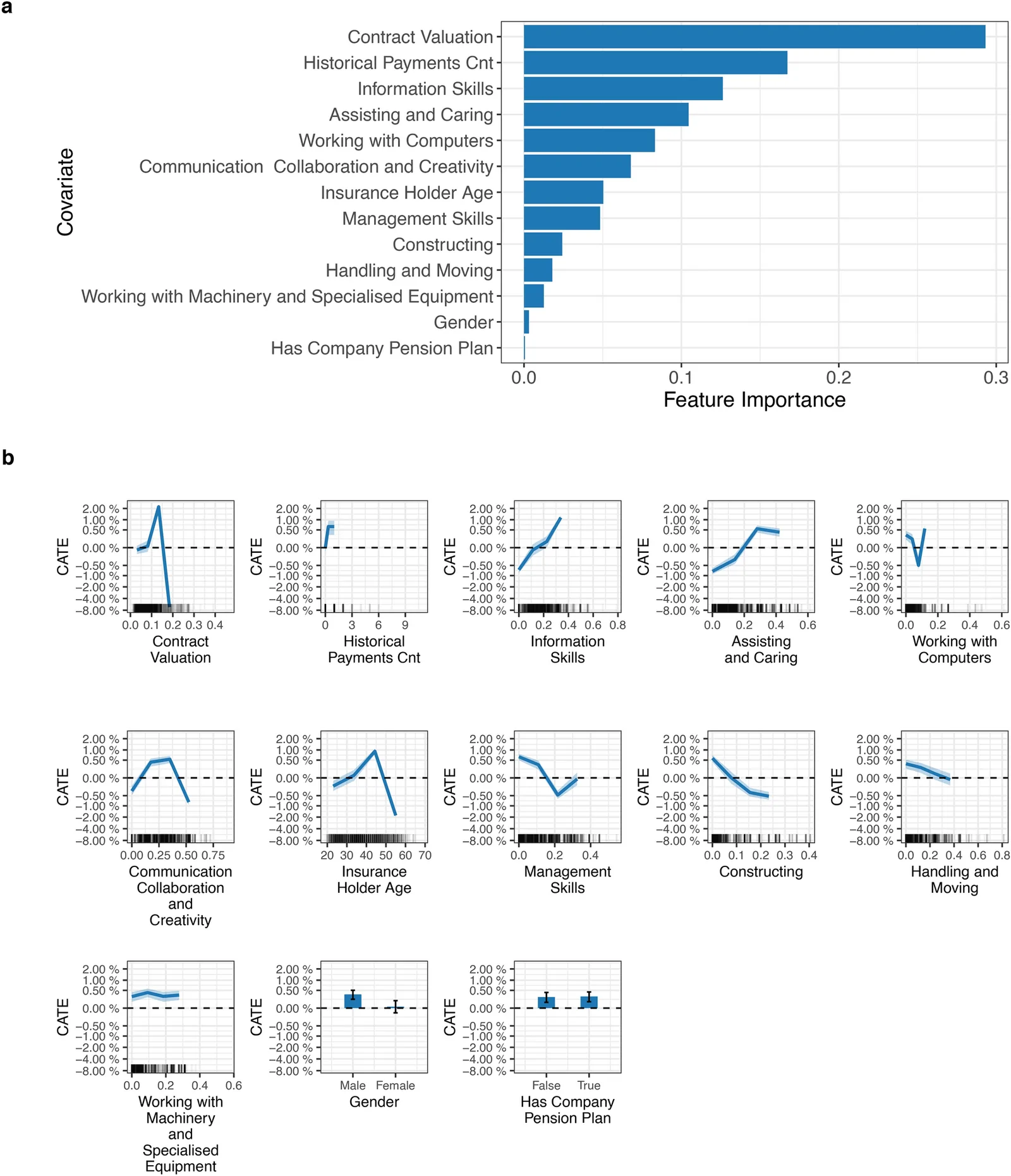
Mechanisms underlying heterogeneous responses to loss- versus gain-framed messages (conversion). This figure uses Explainable AI (XAI) techniques to identify drivers of treatment effect heterogeneity^63^. (**a**) Feature importance across covariates included in the causal forest model, measured as the relative frequency with which a given covariate is used to split observations across trees. (**b**) Partial dependence plots^65^ among covariates showing the marginal effect of a given feature on the predicted CATE, while averaging over the distributions of all other features. A positive estimated CATE is associated with a higher probability of converting under loss framing than under gain framing. The uncertainty bands represent 95% confidence intervals. Rug plots below each panel show the distribution of observations. The line-charts are truncated at the 5th and 95th percentiles to avoid extrapolation beyond the region of common support at the distribution tails. Discrete covariates are represented as bar-charts with 95% confidence intervals. The y-axis is on a pseudo-log transformed scale.

We start by assessing feature importance in the causal forest, here measured as the relative frequency with which a given covariate is used to split observations across different branches of the trees in the causal forest (Fig. 4a). In this context, a covariate is considered important if it repeatedly helps partition the data into subgroups that exhibit systematically different treatment effects. The results indicate that variables capturing customers’ prior saving behavior (i.e., total contract valuation and number of historical extra deposits) are the most influential source of heterogeneity, followed by variables describing the skills required in their current job (i.e., information processing skills, assisting and caring skills, computer skills).

To understand how these key characteristics shape responsiveness to message framing, we next examine partial dependence plots^65^, which visualize the relationship between individual covariates and the estimated CATE while averaging over all other characteristics (Fig. 4b). These plots allow us to interpret the direction and functional form of heterogeneous framing effects. For example, loss-framed messages are more effective at nudging additional contributions among customers who hold high-value policy contracts and work in jobs that require high information-processing or computer skills. Conversely, gain-framed messages are more effective for customers who have made frequent extra deposits in the past, who are older than 50 years, or who work in jobs requiring high management skills. Moreover, many of the dependence patterns are non-linear. For example, the effectiveness of loss-framed messages rises gradually with age until 50 and then drops sharply. Such non-linear effects highlight a key advantage of Causal ML, which is able to estimate flexible effects without imposing strong parametric assumptions^50^..

As a robustness check, we computed SHAP-based explanations^66^. Specifically, we approximated Shapley values for conditional treatment effects and obtained global importance rankings (see Figure S5 in the Supplementary Materials). The resulting SHAP-based importance patterns were qualitatively consistent with those obtained from above.

### Heterogeneous effects on the intensive margin (saving amount)

#### Heterogeneity analysis

**Fig. 5:**
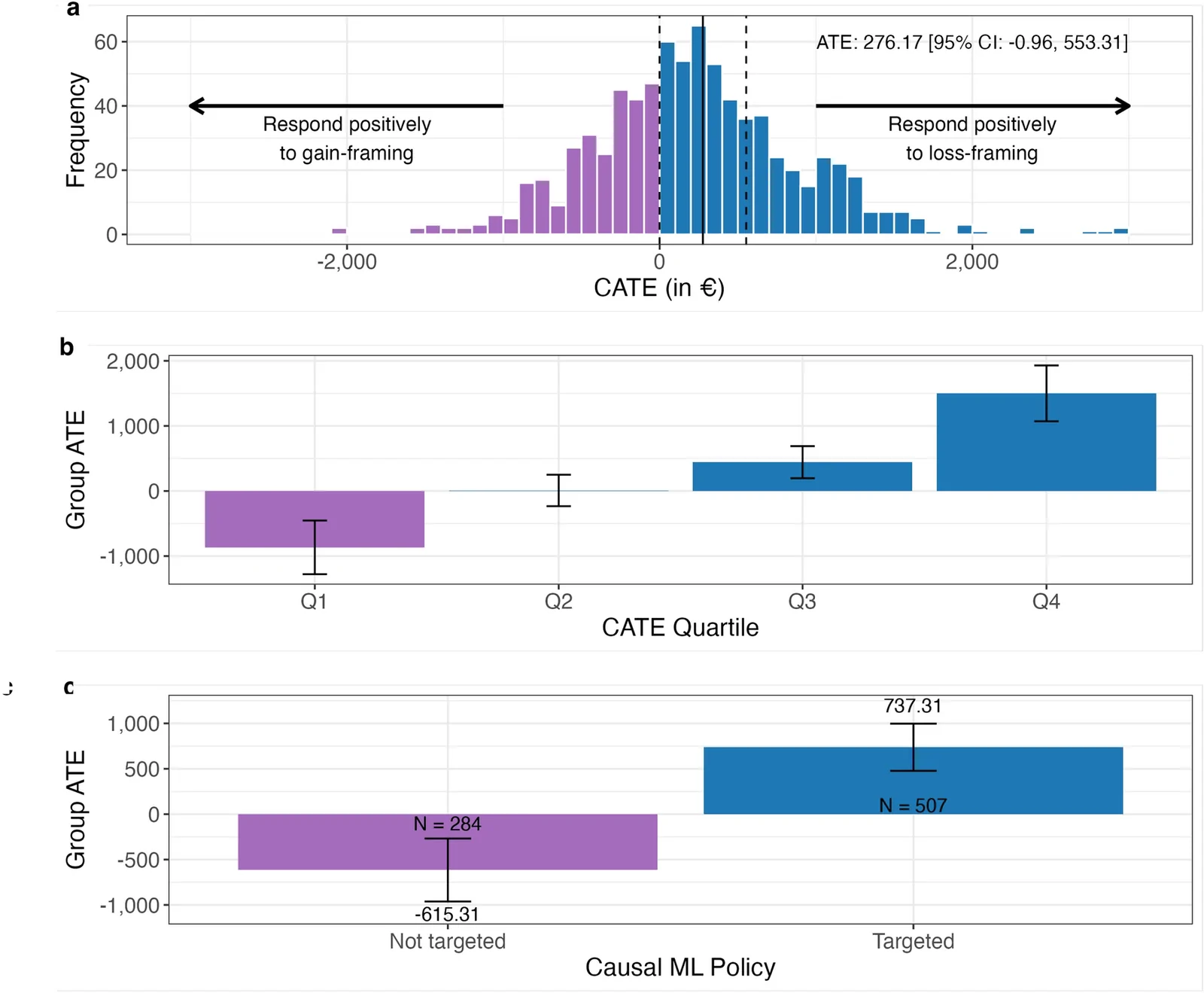
Heterogeneous effects of loss versus gain framing on savings amount (intensive margin). This figure shows heterogeneity in the effect of message framing on customers’ retirement savings amounts among those who make an additional contribution, based on a causal forest that was trained on data from Field Experiment 1 and evaluated out-of-sample using data from Field Experiment 2. (**a**) Distribution of CATEs of sending a loss-framed message (versus a gain-framed message) on savings amount. (**b**) ATE of sending a loss-framed message on the savings amount aggregated by CATE quartiles. (**c**) Evaluation of a Causal ML–based targeting policy. The uncertainty margins depicted in the plots represent 95% confidence intervals.

For the savings amount (intensive margin), the distribution of CATEs again reveals substantial heterogeneity in how individuals adjust their savings amounts in response to loss- versus gain-framed messages (Fig. 5a). Positive CATE values identify customers who contribute higher amounts under loss framing than under gain framing, whereas negative CATE values identify customers for whom loss framing reduces contribution levels relative to gain framing. For the converted participants in the validation experiment (i.e., *N=791* of the $N=18{,}288$ in Experiment 2; the forest is trained on $N=1{,}195$ of Experiment 1), the CATEs ranged from €−2,052 to €2,969 (first to third quartile: €−196 to €595). Approximately 64% of customers exhibit positive CATEs, implying that loss framing increases their contribution amounts, while 36% exhibit negative CATEs and thus respond more favorably to gain framing. For those customers with increases in contributions due to loss framing, the average contribution is €737, while, for those where loss framing backfires, the decrease in contribution is €615 on average. Further, 29% of customers experience even an increase in savings amounts of at least €500 under loss framing, and 13% experience increases exceeding €1,000. In contrast, for 12% of customers, loss framing reduces savings amounts by more than €500. These patterns highlight that framing effects on the intensive margin are not only heterogeneous but also large relative to the typical contribution (€1,868).

To formally test for heterogeneity, we again compute the targeting operator characteristic (TOC; see Figure S4 in the Supplementary Materials) and the corresponding rank-weighted average treatment effect (RATE). The RATE test confirmed the presence of heterogeneous effects (€665, 95% CI: €306, €1,025).

#### Robustness checks

To assess the robustness of the estimated CATEs, we again examine whether sorting customers by their predicted CATEs yields systematically different realized treatment effects across groups. The distribution of ATEs across CATE quartiles (Fig. 5b) indicates good calibration. The monotonic increase in ATEs across quartiles indicates that the causal forest model successfully ranks customers by their responsiveness to loss versus gain framing along the intensive margin.

Evaluating a Causal ML-based targeting rule that assigns loss-framed messages to customers with positive predicted CATEs and gain-framed messages to all others yields the following outcomes on the hold-out data: For individuals whom the model identifies as responsive to loss framing, the estimated ATE of loss-framed messages on savings amounts is positive and statistically significant (€737; 95% CI: €476, €998; see Fig. 5c). In contrast, for the remaining customers, loss framing has a negative average effect on savings amounts (€−615, 95% CI: €−962, €−268). Together, these results confirm that the causal forest meaningfully distinguishes between customers for whom loss framing increases contribution levels and those for whom it backfires, and that this heterogeneity can be directly exploited for effective personalized message assignment.

#### Drivers of heterogeneity

**Fig. 6:**
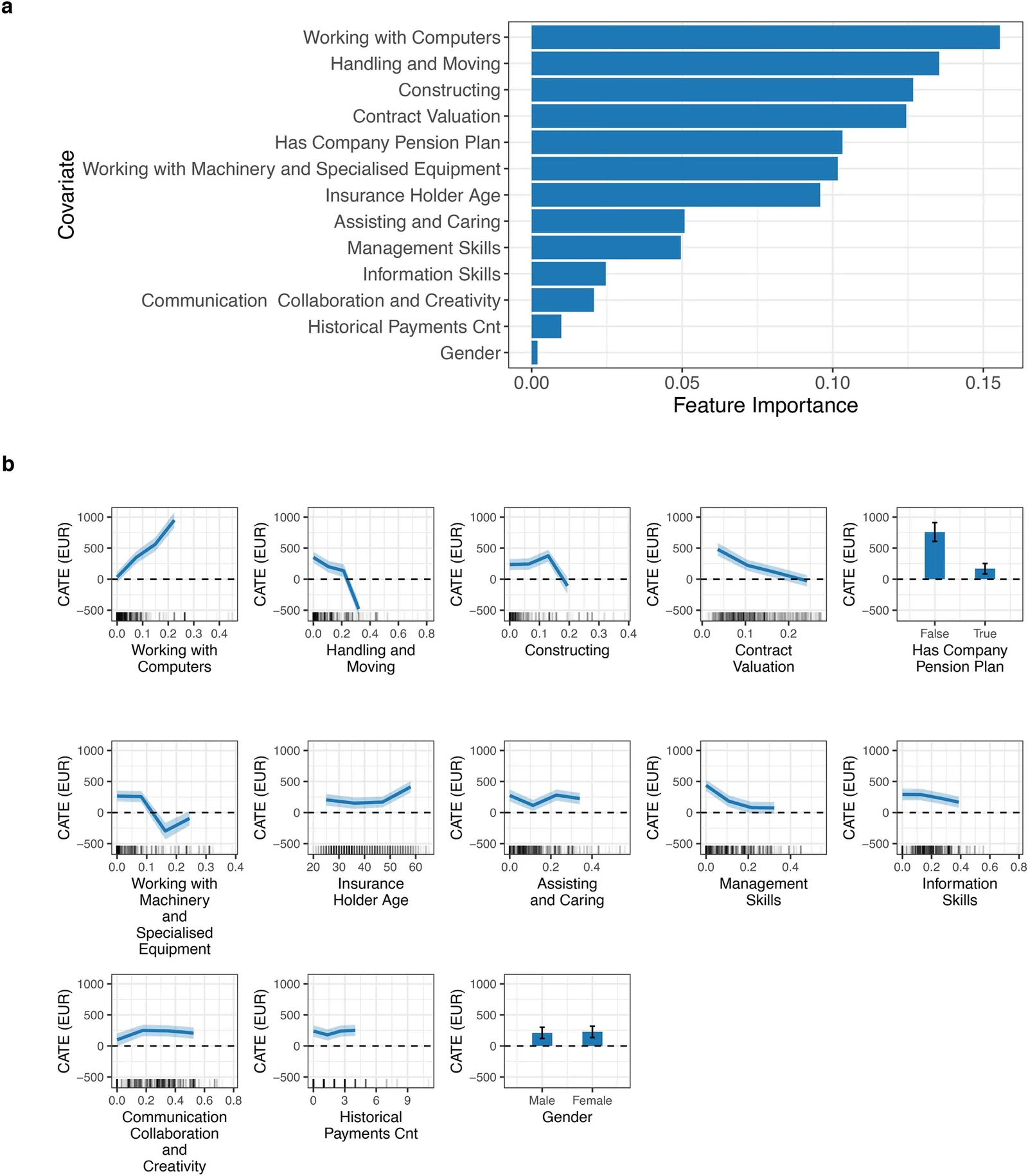
Mechanisms underlying heterogeneous responses to loss- versus gain-framed messages (savings amount). This figure uses Explainable AI (XAI) to interpret the causal forest and identify the main drivers of heterogeneity in framing effects. (**a**) Feature importance across covariates. (**b**) Partial dependence plots showing how the predicted CATE varies with individual covariates. The uncertainty bands depicted in the plots represent 95% confidence intervals. Rug plots below each panel show the distribution of observations. The line-charts are truncated at the 5th and 95th percentiles to avoid extrapolation beyond the region of common support at the distribution tails. Discrete covariates are represented as bar-charts with 95% confidence intervals.

The main drivers of the heterogeneous effects of loss framing on savings amount are job-related variables (see Fig.6a). The partial dependence plots (Fig. 6b) reveal several systematic patterns. Loss-framed messages are more effective for individuals with jobs requiring high computer skills and high communication, collaboration & creativity skills. These are also more effective for customers over 50 years old. In contrast, the higher the contract valuation, the less effective are loss-framed messages. Similarly, loss-framed messages backfire for customers who work in jobs requiring handling & moving, and constructing skills. Again, robustness checks using SHAP-based explanations show qualitatively consistent patterns (see Figure S6 in the Supplementary Materials).

### Benchmark against traditional parametric methods

To assess the value added of Causal ML over traditional statistical approaches, we benchmark the causal forest against parametric models that make a serious attempt to capture effect heterogeneity, following the comparative approach of Baiardi and Naghi^50^. Specifically, we estimate fully saturated probit (extensive margin) and OLS (intensive margin) models on Field Experiment 1, in which the treatment indicator is interacted with all 13 covariates used in the causal forest (see Section “Parametric benchmark”). In-sample, a joint test does not reject the null of homogeneous treatment effects on either margin (extensive: $\chi ^2(13) = 10.39$, *p = 0.662*; intensive: *F(13, 1167) = 1.19*, *p = 0.284*), and no individual interaction remains significant after Holm correction for multiple testing (see Supplementary Table S7). Notably, the interactions with the smallest unadjusted *p*-values—information processing and assisting-and-caring skills on the extensive margin, and handling-and-moving and construction skills on the intensive margin—involve those covariates that the causal forest also identifies among the most important drivers of heterogeneity (Figures 4a and 6a). The parametric specification thus detects traces of the same underlying signal but lacks the flexibility to recover it fully, as it imposes linearity on interactions that the partial dependence plots reveal to be strongly non-monotone.

We further compare both methods out-of-sample on identical evaluation criteria. We use the saturated models fitted on Field Experiment 1 to predict individual-level treatment effects for Field Experiment 2 and evaluate the resulting ranking with the same procedures as for the causal forest. The parametric and causal forest-based rankings differ substantially (Spearman rank correlation of 0.12 for conversion and 0.43 for saving amount). While the parametric ranking is not uninformative—realized treatment effects increase across its CATE quartiles—its RATE is statistically indistinguishable from zero on both margins (conversion: 0.76, 95% CI: *-0.23*, 1.74; saving amount: €285, 95% CI: −€42, €612), whereas the causal forest achieves significant rank-weighted gains on both (2.59 and €665, respectively). The advantage of Causal ML in our randomized setting thus lies not in estimating the average effect, which is identified by design in our randomized experiments and for which all methods agree, but in detecting and exploiting treatment effect heterogeneity without pre-specifying its functional form. The economic consequences of this difference are quantified in the off-policy evaluation below.

### Economic impact of personalized targeting policy

To evaluate the potential economic impact of personalized message framing, we conduct an off-policy evaluation^53^ of a CATE-based targeting policy relative to alternative message framing policies (Table 1). Using randomized assignment and estimated treatment effects, this approach allows us to infer how outcomes would change if each policy were implemented counterfactually. All analyses are performed on the hold-out test set, that is, data from Field Experiment 2 ($N = 18{,}288$ for conversion and *N = 791* for savings amount). For comparison, the observed conversion rate in Field Experiment 2 under randomized assignment of loss and gain messages was *4.33%*, corresponding to 791 customers who decided to make an additional retirement savings contribution. If all customers had received a loss-framed message, the implied conversion rate would be *4.53%*, corresponding to 829 conversions. In contrast, if all customers had received a gain-framed message, the conversion rate would decrease to *4.12%*, corresponding to 753 expected conversions.

**Table 1:** Off-policy evaluation of personalized message framing policies on conversion (extensive margin).

Policy	Subgroups	Treatment	*N*	IP (Conversion)	#Conversions
Random	-	Random	18,288	0.0433	791
All loss	-	Loss	18,288	0.0453	829
All gain	-	Gain	18,288	0.0412	753
By gender	Female	Loss	7,480	0.0417	783
	Male	Gain	10,808	0.0435	
By contract value	Below Median	Loss	9,341	0.0290	798
	Above Median	Gain	8,947	0.0589	
By Mgmt. skills	Below Median	Loss	9,043	0.0452	817
	Above Median	Gain	9,245	0.0442	
Parametric policy	Param+	Loss	8,638	0.0678	867
	Param-	Gain	9,650	0.0291	
**Causal ML-based policy**	**CATE+**	**Loss**	**9,910**	**0.0836**	**1,255**
	**CATE-**	**Gain**	**8,378**	**0.0509**	

The table reports estimated conversion probabilities and expected numbers of conversions under different policies for assigning loss- versus gain-framed messages to individuals. The evaluation was conducted using the hold-out sample from Field Experiment 2 ($N = 18{,}288$). The estimates are obtained via off-policy evaluation^53^, combining randomized assignment with estimated treatment effects to infer how outcomes would change if each policy were implemented counterfactually. We compare uniform assignment rules (random, all loss, all gain), theory-driven subgroup policies based on gender, contract valuation (a proxy for wealth), and management skills (a proxy for financial literacy), a parametric policy derived from the saturated interaction model, and a CATE-based targeting policy derived from the causal forest. The two model-based policies assign loss-framed messages to individuals with positive predicted treatment effects and gain-framed messages to those with negative predicted effects. The Causal ML-based policy yields the highest number of expected conversions, highlighting the economic value of personalization based on heterogeneous treatment effects.

To estimate outcomes under the Causal ML-based targeting policy, we assign loss-framed messages to customers with positive predicted CATEs and gain-framed messages to those with negative predicted CATEs. Using off-policy evaluation, this policy implies an estimated conversion rate of 8.36% among customers predicted to benefit from loss framing ($N = 9{,}910$) and 5.09% among those predicted to benefit from gain framing ($N = 8{,}378$), which would result in 1,255 expected conversions. In the absence of a CATE-based policy, the most successful policy is a uniform loss frame assignment. This is also the assignment that prospect theory predicts to be most effective, as losses loom larger than equivalent gains^23^. The uniform loss frame message therefore serves as a benchmark: it is both the theoretically predicted choice and the economically rational policy of the company in the absence of CATE-based options, and thus the most conservative comparison. In sum, the off-policy evaluation indicates an increase in conversion (extensive margin) from 4.53% under uniform loss framing to 6.86% under the Causal ML-based policy (51% increase).

For comparison, we also estimate conversion rates for alternative targeting rules. These include uniform assignment rules (random, all loss, all gain), theory-driven subgroup policies based on gender, contract valuation (as a proxy for wealth), and management skills (as a proxy for financial literacy), and a parametric policy that assigns frames based on the individual-level effects predicted by the saturated interaction model (see Section “Benchmark against traditional parametric methods”). As shown in Table 1, none of the theory-derived targeting rules outperforms the uniform “all loss” policy (829 conversions). The parametric policy is the strongest alternative, with 867 expected conversions, but still captures only a small share of the attainable gains. In contrast, the Causal ML-based targeting policy yields 1,255 expected conversions, corresponding to a 51% increase relative to the best uniform policy (i.e., “all loss”) and a 45% increase relative to the parametric policy, which highlights the economic value of individual-level personalization based on Causal ML for message framing.

We apply the same approach to compare the economic impact of different policies with respect to savings amounts (see Table 2). In this case, the Causal ML-based targeting policy is expected to generate total retirement savings contributions among the 791 converted customers in the hold-out set of €1,931,571. The runner-up is again the parametric policy (€1,785,426), followed by the best theory-driven policy (targeting by contract valuation, €1,727,373). The causal forest policy thus implies a relative increase of approximately 8% over the parametric policy and of approximately 12% over the best theory-driven policy. Compared to uniform loss framing, the estimated total savings rise from €1,631,150 to €1,931,571, a relative increase of approximately 18%.

**Table 2:** Off-policy evaluation of personalized message framing policies on savings amounts (intensive margin).

Policy	Subgroups	Treatment	*N*	Savings/customer	Total savings
Random	-	Random	791	€2,023.82	€1,600,840.04
All Loss	-	Loss	791	€2,062.14	€1,631,149.58
All Gain	-	Gain	791	€1,981.10	€1,567,046.15
By Gender	Female	Loss	310	€2,226.78	€1,611,080.96
	Male	Gain	481	€1,914.31	
By Contract Valuation	Below Median	Loss	436	€2,778.73	€1,727,373.47
	Above Median	Gain	355	€1,453.09	
By Management Skills	Below Median	Loss	403	€2,392.49	€1,701,520.93
	Above Median	Gain	388	€1,900.38	
Parametric Policy	Param+	Loss	541	€2,555.62	€1,785,426.25
	Param-	Gain	250	€1,611.34	
**Causal ML-based Policy**	**CATE+**	**Loss**	**507**	**€2,559.55**	**€1,931,570.82**
	**CATE-**	**Gain**	**284**	**€2,231.97**	

The table reports estimated savings per customer and total savings under different policies for assigning loss- versus gain-framed messages to individuals. The evaluation was conducted using the converted customers of the hold-out sample from Field Experiment 2 (*N = 791*). The estimates are obtained via off-policy evaluation^53^, combining randomized assignment with estimated treatment effects to infer how outcomes would change if each policy were implemented counterfactually. We compare uniform assignment rules (random, all loss, all gain), theory-driven subgroup policies based on gender, contract valuation (a proxy for wealth), and management skills (a proxy for financial literacy), a parametric policy derived from the saturated interaction model, and a CATE-based targeting policy derived from the causal forest. The two model-based policies assign loss-framed messages to individuals with positive predicted treatment effects and gain-framed messages to those with negative predicted effects. The Causal ML-based policy yields the highest total savings, highlighting the economic value of personalization based on heterogeneous treatment effects.

### Discussion

Our study provides field evidence on how loss versus gain framing influences actual retirement savings contributions. Across two large-scale randomized experiments involving more than 41,000 users of a European app-based retirement savings platform, we find that loss- and gain-framed reminders produce similar *average* effects on both the decision to save (extensive margin) and the savings amount (intensive margin). However, this aggregate, population-level view masks substantial *individual-level* heterogeneity. We find that some individuals are more likely to save under loss framing, while loss framing backfires for others, who, instead, respond positively to gain framing. A personalized targeting rule based on estimated individual-level CATE substantially outperforms uniform framing strategies when evaluated on hold-out data; the off-policy evaluation indicates (i) an increase in conversion (extensive margin) over a uniform loss-framing policy by 51% and (ii) an increase in average contributions among converters by 18%. These results indicate that personalized framing can yield meaningful gains over uniform strategies in this setting.

Our analysis also benchmarks CATE-based framing policies against literature-derived alternatives, showing that these strategies are less effective than individualized targeting. A uniform targeting strategy—such as always applying loss framing based on loss aversion^23^—performs worse than the personalized targeting strategy based on the CATE. Likewise, theory-derived heuristics that assign frames based on simple demographic criteria (e.g., emphasizing losses for females or gains for affluent clients^33–35,38^) deliver only marginal improvements over uniform policies and, in some cases, systematically mismatch frames with individuals. These comparisons reveal that theoretically-informed, but univariate, segmentation approaches do not adequately capture the heterogeneity in the responsiveness to behavioral interventions in retirement savings decisions.

Even a fully saturated parametric interaction model, the strongest non-ML alternative we evaluate, captures only a small share of the gains from Causal ML-based targeting. The parametric policy outperforms the uniform and theory-driven rules, yet yields 867 versus 1,255 expected conversions and €1,785,426 versus €1,931,571 in total savings. Combined with the fact that Causal ML-based policies yield substantial gains in conversions and contributions, this suggests that individuals’ reactions to behavioral interventions are complex, non-linear functions involving interactions among individual-level covariates. Accordingly, the combination of covariates and a non-linear form^50^ allows to understand previously not visible heterogeneous effects.

#### Theoretical contributions

Our study contributes to research on behavioral interventions in the context of retirement savings. Earlier work demonstrates that defaults and commitment nudges can shape saving behavior, most prominently through the automatic enrollment^16^ and “Save More Tomorrow” programs that raise contributions over time^15^. Subsequent studies show that behavioral interventions can influence participation and contributions, including contribution-rate nudges in employer communications^67^, framing and assurance messages in pension interfaces^38^, social-norm messages embedded in retirement products^68^, and family-security reminders in retirement savings^69^. Yet, these studies typically report *average* effects and treat savers as if they respond uniformly. Our results show that the effectiveness of loss- and gain-framed messages can look similar on average, but may produce substantial *heterogeneous* effects at the individual level.

Our Explainable AI analyses further capture the drivers behind the observed effect heterogeneity. We show that heterogeneous treatment effects arise through a constellation of behavioral, socioeconomic, and occupational characteristics—including prior saving behavior, the economic value of existing contracts, age, income, and job-related skill profiles such as information processing or computer skills. These results suggest that heterogeneity is neither idiosyncratic nor random: loss- and gain-framed messages act through interactions and non-linear effects that differ across individuals. By revealing these patterns, we provide starting points for theorizing about potential mechanisms underlying framing effects in complex financial contexts, which warrant systematic investigation in future research. Moreover, the observation that effect heterogeneity varies with the dependent variable under consideration—conversion (the extensive margin) versus contributed amounts (the intensive margin)—indicates that the heterogeneous effects of loss versus gain framing depend not only on the general alignment between message and recipient, but also on distinct phases of the customer decision-making process.

We contribute to behavioral science by showing the importance of heterogeneity in human behavior. As such, our study reveals the limitations of existing field experiments that evaluate behavioral interventions primarily through population-wide average effects. While informative, this perspective focuses mainly on only “whether” an intervention works “on average”, rather than “for whom”, and can therefore obscure substantial heterogeneity in responses, as individuals may react very differently to the same intervention^39^. As a result, interventions that exhibit weak or null average effects can nonetheless generate large positive effects for some individuals and negative effects for others, which cancel out in aggregate statistics^41,69–72^. By combining randomized field experimentation with Causal ML, we show how modern, nonparametric estimation tools can reveal complex, nonlinear patterns of human responses to behavioral interventions in a data-driven way.

Beyond economic impact, our findings also raise distributional and algorithmic fairness questions about who benefits from personalized message targeting. A policy that optimizes solely for additional savings volume will naturally prioritize individuals with higher contract valuations and greater capacity to increase contributions, which risks systematically overlooking customers with lower wealth, lower financial literacy, or more constrained budgets. Such disparities can arise even in the absence of intentional exclusion, as algorithmic decision systems that optimize aggregate or overall performance tend to prioritize majority or high-response groups^73^ and may systematically disadvantage historically underserved populations unless fairness considerations are explicitly incorporated into the optimization objective^74,75^. Such targeting may therefore reinforce existing inequalities in retirement savings, even as it improves aggregate outcomes. By separately modeling extensive and intensive margins, our approach already highlights that participation (extensive margin) and contribution size (intensive margin) can be optimized differently, and illustrates how one might design targeting rules that place greater weight on supporting underserved groups into the savings system rather than exclusively maximizing total volume.

#### Possible mechanisms

Although the identification of mechanisms is not the primary goal of this study, several of the effect moderators we identified correspond to mechanisms discussed in prior research. We discuss the three most important moderators in turn: wealth, prior saving behavior, and occupational skills.

Wealthier customers react more strongly to loss framing, a pattern that prospect theory predicts. The reminder presents the same choice, namely whether to make an additional retirement savings contribution before the end of the tax year, either to secure tax advantages and additional retirement provision or as to lose them. Loss aversion predicts stronger reactions when the decision is coded as a loss^23^, and this pull should increase with the stakes involved. Under a progressive tax system such as Switzerland’s, the tax saving attached to an additional retirement contribution rises with income and wealth, so leaving the contribution allowance unused means giving up more for wealthier customers. Loss aversion is also particularly pronounced among high-income taxpayers, who face more complex tax situations and have more options to react^76^. Figure 4b shows exactly this pattern: loss framing becomes more effective as contract valuation increases. The two ends of the distribution deviate from this pattern. Because top marginal tax rates are capped, the tax saving from contributing stops growing at the highest wealth levels, which may explain why the effect reverts at the very top of the distribution^77^. Conversely, for resource-constrained individuals, Beshears et al.^18^ have shown that unattainable contribution goals can reduce uptake, which matches the lower bound in Figure 4b.

Customers who have frequently made additional payments in the past respond better to gain framing, and two mechanisms are consistent with this pattern. First, individuals with substantial experience in a decision exhibit weaker endowment effects and behave more in line with neoclassical rather than prospect-theoretic predictions^78,79^. Frequent past contributors are precisely the customers with the most experience in retirement savings decisions, suggesting that the effect of loss framing weakens with repeated exposure to it. Second, regulatory focus theory predicts that messages persuade when their frame fits the recipient’s motivational orientation: gain frames are more persuasive for promotion-oriented individuals, whereas loss frames fit a prevention orientation^80^. Customers who repeatedly and voluntarily add contributions are plausibly promotion-oriented, approaching retirement saving as growth toward a goal rather than as protection against a shortfall, so gain frames fit their orientation better.

For occupational skills, loss framing is more effective for customers in jobs requiring high information processing and computer skills. The loss frame points to an outcome that is never directly experienced; what a customer gives up by not contributing, that is, the tax saving and the additional retirement provision, must be mentally constructed from the quantified information in the reminder. Customers with strong information processing skills are better able to translate the stated amounts into these concrete consequences^81^, and highly numerate individuals derive stronger affective reactions from such numerical information^82^. Because losses loom larger than equivalent gains^23^, a more concretely perceived consequence amplifies the response to the loss frame more than to the gain frame, so the framing effect widens with information-processing ability.

The heterogeneity uncovered by Causal ML is thus not idiosyncratic but in line with established behavioral theory. A systematic investigation of these mechanisms remains a task for future research.

#### Implications and limitations

Finally, our study has practical implications for the design of behavioral public policies by showing that theory-derived, uniform interventions—such as always using loss framing in line with loss aversion, or segmenting individuals using coarse demographic heuristics—tend to underperform compared to individualized, data-driven personalization rules. Our findings thus point to the value of complementing average-based behavioral interventions with more individualized approaches that respect individual variation in psychological responses. The covariates underlying the personalized policy (demographics, contract characteristics, prior saving behavior, and standardized occupational skill profiles) are recorded in the operational systems of many FinTech and e-commerce companies, so comparable targeting rules are feasible without additional data collection. Feasibility, however, is not sufficiency. The effectiveness of any such policy depends on the quality of the underlying CATE estimates, and our results show the value of personalization conditional on this covariate set and model. Richer data, such as measures of financial literacy or risk preferences, could yield further gains. Any deployment should validate the estimated policy out-of-sample, as we do here.

Several features of this study strengthen the validity and reliability of our findings. First, the interventions were delivered within a real-world financial system and captured economically meaningful, high-stakes behavior rather than hypothetical choices or intentions. Second, the large sample sizes in the field experiments provide substantial statistical power, which is beneficial for detecting heterogeneous treatment effects across subgroups. Third, by separately analyzing the extensive and intensive margins of saving behavior, we show that loss versus gain framing does not operate uniformly across decision stages. Fourth, we use the causal forest algorithm^47,48^, an established method from Causal ML to uncover treatment-effect heterogeneity. A strength of causal forests is that they do not impose restrictive linearity or additivity assumptions. Rather, the causal forest allows us to capture complex, non-linear relationships and high-dimensional interactions that would be difficult to detect using conventional subgroup analyses or linear interaction models, which typically require strong a priori assumptions about where heterogeneity lies. Fifth, we train the Causal ML model on one large-scale randomized field experiment and evaluate it on a second, *independent* experiment. This two-experiment design provides a strong test of external validity and generalizability for heterogeneous treatment effect estimates^54–56^.

Of course, our study is not without limitations. First, our sample consists of individuals who are already enrolled in a retirement savings product, which may limit the generalizability of the findings to other populations, such as individuals who are not yet engaged in retirement savings. Second, the deployment of the behavioral intervention in the field did not allow us to pre-register the experiment nor share the data openly to protect the privacy of customers. Third, we did not take full advantage of Causal ML in our randomized study. Two commonly cited advantages of Causal ML are flexible and high-dimensional adjustment for confounding^50^; both are largely inapplicable in our cases, because our data stems from randomized experiments and contains only a dozen variables. Hence, it is not surprising that the estimated average treatment effects agree over all estimators. The main value added of Causal ML in our cases lies in the detection of effect heterogeneity: the causal forest identified non-monotone effect modification that the linear interaction models did not detect. Finally, some limitations of Causal ML warrant caution. Causal forests provide no closed-form interpretation, so the insights on heterogeneity drivers rely on post-hoc explainability tools that are descriptive in nature. Individual CATE estimates are regularized and noisy—the tails of the estimated CATE distribution are wide relative to the interquartile range—so individual-level point estimates should not be over-interpreted. Causal ML results can also be sensitive to hyperparameter and sample-splitting choices; we used the package’s default hyperparameters, with honest splitting configured following guidance for imbalanced data rather than tuned values. Reliable heterogeneity estimation requires large samples, which constrains the analysis of the intensive margin, where only 1,986 customers converted. These limitations notwithstanding, the out-of-sample replication of both the CATE ranking and the targeting-policy gains indicates that the detected heterogeneity is systematic rather than an artifact of flexible estimation.

Overall, this study demonstrates that the effectiveness of loss versus gain framing in retirement savings cannot be assessed solely by which frame performs better on average, but this requires understanding who responds to which frame. Recognizing and systematically analyzing this heterogeneity can inform more precise behavioral theory and the design of more effective policies to strengthen financial security in aging societies.

### Methods

#### Experiment overview

We study whether the framing of annual reminder messages affects retirement savings behavior. Specifically, we test whether presenting information about remaining tax-deductible contribution capacity as a potential loss versus a potential gain influences customers’ decisions to make additional retirement savings contributions. To do so, we partnered with a European FinTech company that offers so-called unit-linked retirement savings products and communicates with customers through a mobile wealth management application. As part of its regular customer communication, the company sends reminder messages once per year informing customers about their remaining tax-deductible contribution amount for the current tax year.

We experimentally varied the framing of these annual reminders in two large-scale field experiments conducted in consecutive years. Field Experiment 1 (2021) included *N=*22,919 customers, and Field Experiment 2 (2022) included *N=*18,288 customers. Both experiments were implemented within the same institutional setting and followed the same experimental logic. The difference in sample size across the two experiments reflects natural variation in the active customer base across years, including account openings, closures, and eligibility changes. Across both experiments, reminder messages were sent once per customer at the beginning of November, a time when customers could still adjust their contributions for the current tax year. We then measured how customers respond to the messages.

Our experiment took place in Switzerland and focuses on pillar 3a retirement accounts, a widely used tax-advantaged private retirement savings vehicle (see, e.g., Bütler (2016)^77^ for an overview of the Swiss pension system). Customers can deduct contributions up to a legally defined annual maximum from their taxable income, creating a clear financial incentive to contribute before the end of the calendar year. Nevertheless, while our empirical context is Swiss, the underlying institutional structure is comparable to tax-advantaged retirement savings systems in many other countries. For example, individuals in the United States can deduct contributions to 401(k) plans or individual retirement accounts (IRAs), and individuals in Germany can deduct contributions to Riester pensions. In all these systems, customers face an annual contribution cap and receive information about remaining deductible capacity, making the framing of such information broadly relevant beyond the Swiss setting.

#### Conditions

Each customer received a reminder through the wealth management app of the FinTech (see Figure 2) and via e-mail at the beginning of November, when customers could still adjust their contributions for the current tax year in order to maximize the tax-deductible amount. Customers could initiate additional contributions directly through the app or via a bank transfer based on the information provided in the reminder. However, the reminders varied in whether loss- versus gain-framed messages were used.

To design loss- and gain-framed messages, we drew on words signaling losses and gains from previous studies. We selected the terms *“losing”*^31,83^ and *“missing out”*^84^ for the loss condition, and *“benefit”*^31,83–85^ and *“secure”*^31,85^ for the gain condition.

In addition to containing numerical data and verbal explanations, the wealth management app in our study contained various visualizations of financial data, allowing for a multimodal framing. For instance, customers saw a vertically stacked bar chart summarizing the already paid amount, a projection of payments until the end of the year, and the remaining tax-deductible amount. For the loss condition, we colored the area of the vertically stacked bar chart signaling the open amount bar in red^86^, while we colored it in silver-gray for the gain-framed message. In all statistical analyses in this paper, the gain-framed condition serves as the control group and the loss-framed condition serves as the treatment.

#### Outcome variables

We measure actual retirement savings behavior rather than self-reported intentions. In particular, our setting allowed us to observe important outcomes with respect to savings behavior, namely, by the (1) *extensive margin* (i.e., decision to save, given by conversion: do customers make an additional payment?) and (2) *intensive margin* (i.e., contribution: how much do converted customer save?). The extensive margin was operationalized via the binary variable *Converted*, which takes the value of 1 for customers who made a payment and 0 otherwise. The intensive margin was operationalized via the continuous variable *SavingsAmount*, which measured the amount saved (in €) after deciding to invest. We observe both outcomes over the same fixed time window; that is, we record all additional contributions made from the date of message delivery until the end of the respective tax year, and we ignore contributions made before message delivery.

#### Procedure

Randomization was implemented at the individual customer level within the partner company’s production system using a pseudo-random number generator. The assignment was not stratified by covariates and did not depend on past behavior or customer characteristics. Customers were unaware that the reminder wording was randomized, and the researchers analyzing the de-identified data did not have access to any information that could identify individual customers.

In Field Experiment 1, participants were allocated in a 2:1 ratio to the loss framing and gain framing conditions, respectively, whereas Field Experiment 2 used a 50:50 allocation. The larger proportion of participants assigned to the loss framing condition in Field Experiment 1 reflected our initial hypothesis, grounded in prospect theory^23^, that loss framing would yield higher conversion rates and larger saving amounts, thereby generating greater sales for the partner company.

#### Study participants

Eligible customers were those with an active pillar 3a account who had not yet reached the legal tax-deductible maximum for the current year and who had consented to receive communications from the FinTech. The first experiment included $N = 22{,}919$ customers and the second $N = 18{,}288$. Both experiments employed a between-subjects design in which customers were randomly assigned to one of two conditions.

The average customer was 36 years old and employed (rather than self-employed), and 41% were female. The average contract value was €88,000 (for easier readability, the contract valuation is reported in million EUR in the following chapters), and each customer made, on average, 0.11 extra payments into their retirement account in the past. Across conditions, the standardized mean differences for all covariates are well below the threshold of 0.1, which indicates good covariate balance between experimental conditions^87^. In addition, the FinTech maintains rich data about the jobs of its customers. Specifically, they match their customers’ job titles with the European Union’s ESCO Skill-Occupation Matrix Tables^88^. This database contains information regarding qualifications and skills required for each job category (see Table S2 for detailed variable descriptions). Summary statistics are in Tables S3 and S4, reported separately by experiment.

The socioeconomic variables are also used as covariates inside the causal forest models. That means, we later use these skill dimensions as covariates to identify heterogeneity in framing effects by type of work. Importantly, all covariates *X* used in the causal forest models are measured prior to the framing intervention.

#### CATE estimation

To estimate the CATE of the two framing conditions on the different outcomes of interest, we follow a standard Causal ML approach^89–91^. Let $Y_i$ denote the outcome for participant *i* (i.e., binary for conversion and continuous for saving amount), and let $T_i \in \{1,0\}$ indicate whether participant *i* was assigned to the treatment (loss framing: *T=1*) or control (gain framing: *T=0*) group. We use $Y_i(1)$ and $Y_i(0)$ to denote the potential outcomes that participant *i* would exhibit under treatment and control, respectively. The individual treatment effect is $Y_i(1) - Y_i(0)$, but it is unobservable because we never see the same participant under both conditions. Instead, we estimate the CATE, which, given a vector *X* of observed individual-level covariates, is defined as

1 $$\begin{aligned} \tau (x) = E [Y(1) - Y(0) \mid X=x] . \end{aligned}$$

Since the treatment in our field experiments was assigned randomly, *Y*(1), *Y*(0), and *X* are independent of *T* so that randomization ensures *(Y(1), Y(0), X) ⊥ T*. This allows us to identify the CATE as the difference in conditional expectations, i.e.,

2 $$\begin{aligned} \tau (x) = E [Y \mid T=1, X=x] - E [Y \mid T=0, X=x] . \end{aligned}$$

Hence, the CATE captures individual-level variation in the response to loss versus gain framing. In our case, we use a broad set of socioeconomic characteristics, including the skill dimensions as covariates *X*: gender, age, employment status, contract valuation, number of historical payments, information skills, computer skills, management skills, communication collaboration creativity skills, assisting and caring skills, construction skills, machinery and equipment skills, handling and moving skills (see Table S2).

To empirically estimate CATEs in our study, we applied the causal forest algorithm^47,90^. The causal forest is a state-of-the-art, nonparametric Causal ML method specifically designed to uncover heterogeneity in treatment effects. The causal forest extends the traditional random forest^92^ to model the CATE across subpopulations. To do so, it recursively partitions the covariate space using splitting rules that maximize treatment effect heterogeneity while controlling variance. We chose the causal forest algorithm due to several key advantages. First, it does not rely on restrictive parametric assumptions and thus allows for the flexible modeling of complex, nonlinear interactions between covariates and treatment effects^47–49^. Furthermore, the ensemble structure of the causal forest supports out-of-sample validation, which we assess through validation in an independent experiment.

In our empirical application, we used Field Experiment 1 as the training dataset and the Field Experiment 2 as the hold-out validation set. Importantly, all reported estimates (i.e., CATE, ATE, RATE) are computed on the hold-out validation set, given models fitted on the training sets. This design allows us to evaluate the performance of the causal forest and the resulting CATE-based targeting policies entirely out-of-sample. By training the model on one large-scale randomized experiment and validating it on an independent experiment conducted at a different point in time, we follow a gold-standard approach to validate subgroup effects^54–56^.

Our implementation of the causal forest makes use of the *grf* package (version 2.4.0) inside *R* (version 4.2.1). We used the default hyperparameter settings of the causal forest function (i.e., num.trees=2000, mtry=13, min.node.size=5, sample.fraction=0.5) and followed guidelines on honest sample splitting for accurate individual-level treatment effects in imbalanced data^93^. Following standard practice in *grf*, we used orthogonalized (doubly robust) scores to estimate CATEs, ATEs, and RATEs. The SHAP analysis was performed using the *fastshap* (version: 0.1.1) and *shapviz* (version: 0.10.2) packages.

#### Robustness checks

To compare the CATEs against the ATE, we estimated the ATE and corresponding confidence intervals using the augmented inverse probability weighting (AIPW) estimator^57,58^. The AIPW estimator is a doubly robust, nonparametric method that is known to be both flexible in capturing nonlinear relationships and interactions and robust to skewed, non-normal, and bounded outcome distributions. We implemented the AIPW estimator using the *grf* package (version 2.4.0). In addition to using an AIPW estimator for estimating the ATE, we also applied more traditional statistical models (see Supplementary Text).

As alternative tests for treatment effect heterogeneity, we computed the *targeting operator characteristic (TOC) curve*. The TOC evaluates whether individuals who are ranked as more responsive by the model indeed exhibit larger realized treatment effects when they are prioritized for treatment, compared to a benchmark of random assignment. In addition, we computed the associated *rank-weighted average treatment effect (RATE)*^59^, which summarizes the area under the TOC curve into a single scalar measure. We implemented the TOC and RATE estimations using the *grf* package (version 2.4.0).

We conducted several further robustness checks. We first applied the effect scores method^60–62^, following best practices in causal machine learning. As a second robustness check, we compared the CATE estimates from our causal forest model to those from a T-learner^94^, which estimates separate outcome models for the treatment and control groups and computes CATEs as their difference. For each dependent variable, we computed Pearson’s correlation coefficient between the CATE estimates from the causal forest and the T-learner, which yielded correlation coefficients of 0.815 (*p*-value *< 0.001*) for the decision to save and 0.833 (*p*-value *< 0.001*) for the saving amount. These high correlations suggest that our results are consistent across different Causal ML estimation strategies.

#### Parametric benchmark

Following Baiardi and Naghi^50^, we benchmark the causal forest against traditional parametric specifications. On Field Experiment 1, we estimate fully saturated models in which the treatment indicator is interacted with all 13 covariates from the causal forest: a probit model for conversion and OLS for the log saving amount among converters. We test for effect heterogeneity via a joint likelihood-ratio (*F*-) test of all interaction terms and adjust individual interaction *p*-values for multiple testing using the Holm procedure. For the out-of-sample comparison, we predict individual-level parametric treatment effects for Field Experiment 2 as the difference between the fitted outcomes under loss and gain framing (on the response scale; for the intensive margin we use a saturated OLS in euro levels so that predictions are on the same scale as the forest CATEs). The parametric predictions are then evaluated with procedures identical to those used for the causal forest: the RATE is computed against the same evaluation forest, quartile calibration uses the same transported group-ATE estimator, and the off-policy evaluation applies the same construction as in Tables 1 and 2. Note that, because treatment was randomized, the average treatment effect is identified by design and no estimator can improve upon it; the comparison therefore targets the detection and exploitation of treatment effect heterogeneity, which is where the two approaches differ.

#### Personalized targeting rule

To assess how well behavioral interventions can be personalized, we follow best practices and use an off-policy evaluation^53^. Formally, we develop personalized targeting rules derived from the estimated CATEs. The optimal policy selects treatment according to

3 $$\begin{aligned} \pi (X) = \arg \max _{T \in \{ 0, 1 \}} \ E[Y|X, T] \end{aligned}$$

where *T=1* denotes loss framing and *T=0* gain framing.

For benchmarking, we also introduce several heuristic targeting rules based on prior literature. In financial decision-making contexts, prior studies identified gender, wealth, and financial literacy as key moderators of the effect of loss versus gain framing. Gender as a moderating factor is widely discussed in the literature^95^, with most studies showing that, on average, women tend to exhibit higher loss aversion and stronger sensitivity to potential financial shortfalls^35,38^. Wealth also shapes responses to loss and gain frames: lower-wealth households typically experience stronger subjective loss aversion, leading us to expect stronger reactions to loss-framed messages among this group^33–35^. To operationalize this moderator, we use contract valuation as a proxy for wealth. Finally, because individuals with lower financial literacy rely more on heuristic processing of monetary information and therefore show stronger loss-averse reactions, we predict that loss framing is particularly effective for customers with lower financial literacy^33,34^. We use management skills as a proxy for financial literacy in our analysis^88^.

## Supplementary Information

Supplementary Information.

## Data Availability

The datasets analyzed in this study are not publicly available because of a non-disclosure agreement and data-protection regulations that protect the personal data of the participants. The data have been made available to the editors and reviewers through the journal’s submission system for the purpose of peer review. All code required to reproduce the analyses is publicly available to all readers at https://github.com/kboesch/causal-ml-loss-gain-framing.
