# Supplementary material for: Causal machine learning reveals heterogeneous effects of loss versus gain framing on retirement savings
Source: Sci Rep. 2026 Nov 2;16:36753. doi: 10.1038/s41598-026-76813-1 (PMC13731136; doi:10.1038/s41598-026-76813-1)
Supplement: Supplementary file 1 [file 41598_2026_76813_MOESM1_ESM.pdf]

Supplementary Materials

Supplementary Text

Figs. S1 to S18

Tables S1 to S7

Supplementary Materials for
Causal machine learning reveals heterogeneous effects of loss versus gain framing on retirement savings

Kevin Bösch, Oliver Müller*, Markus Weinmann, Stefan Feuerriegel

*Corresponding author. Email: oliver.mueller@uni-paderborn.de

This PDF file includes:

Supplementary Text

Figures S1 to S19

Tables S1 to S7

Supplementary Text

Related work

Table S1 shows an overview of the existing experimental studies on loss versus gain framing in financial decision-making. These studies vary in (1) contexts (e.g., retirement savings, investment choices, credit card payments), (2) cues to frame the message (i.e., text or color), (3) dependent variables (i.e., intention, extensive margin, or intensive margins), (4) research designs (lab versus field), and (5) findings of the study.

The overview shows that most studies were conducted in the lab and measured intentions rather than actual decisions (i.e., extensive or intensive margin). Prior research has shown that intentions correlate with behavior but are not identical, pointing to a potential intention–behavior gap [33]. In our study, we therefore focused on actual behavior, namely whether customers invested ("extensive margin") and how much they invested ("intensive margin").

The literature also reveals heterogeneous effects of framing. For example, framing effects are moderated by gender [35, 36, 33], wealth [34, 35, 33] and financial literacy [37, 33, 34]. In particular, lower financial literacy is associated with stronger framing effects [33, 34]. Similarly, less financial freedom (i.e., lower wealth or higher credit card debt) is associated with increased framing effects [33, 34, 35]. Regarding the heterogeneous effects of gender, some studies [33] find that men are generally more susceptible to framing than women, but women are more affected by loss framing than men [38, 36]. Finally, context matters: Bockweg et al. (2018) [35] found that certain subtypes of framing (consumption- vs. investment-oriented framing) interact with gender [95]. In addition to sociodemographic heterogeneity, Cassotti et al. (2012) [96] showed that participants' emotional state matters. When participants were exposed to positive emotions (i.e., by showing them pictures that induced positive emotions), the effect of loss framing was no longer observable. Similarly, the study by Yoon & La Ferle [97] highlights the complex interactions among effects among individuals. They show that self-orientation-loss-frames work better or worse depending on the personality (i.e., better for lower level of collectivism) of the individual. Together, these studies show that framing effects are not uniform but depend on individual characteristics. This evidence motivates our use of causal ML to identify and quantify heterogeneous effects of framing.

Table S1: Experimental studies on loss/gain framing in the context of financial decision-making. Main difference is that prior literature focuses on the ATE (or a coarse subgroup ATE), while our work aims to estimate high-dimensional CATE to understand individual-level treatment heterogeneity.

**Source**	**Context**	**Outcome**	**Design**	***N***	**Estimand**	**Findings**
Bateman et al., 2015 [37]	Investment choice	Intention	Lab	1,199	ATE	Presenting downsides (i.e., loss-framed) outperformed positive framing. The effect was moderated by financial literacy.
Bockweg, et al., 2018 [35]	Retirement saving	Intention	Field	2,368	ATE	Loss-framed messages led to higher lump sum and more risk taking. The effect was moderated by gender, wealth, and whether the frame was consumption- vs. investment-oriented.
Brown et al., 2016 [33]	Retirement saving	Intention	Lab	1,437	ATE	Gain framing led to earlier claiming age. The effect was moderated by gender, financial literacy, and wealth.
Cassotti et al., 2012 [96]	Investment choice	Extensive Margin	Lab	57	ATE	Loss framing induced more risk-seeking behavior. Emotional context moderated the susceptibility to loss framing.
Cho et al., 2014 [98]	Retirement saving	Intention	Lab	461	ATE	Null result
Eberhardt et al., 2021 [38]	Retirement saving	Intention	Lab	85 & 340	ATE	Loss framing increased ad effectiveness. Loss aversion moderated the effect of assurance frames.
Faralla et al., 2017 [36]	Retirement saving	Intention	Lab	135 & 241 & 68	ATE	Loss framing led to later payout. The effect was moderated by gender.
Ganzach & Karashi, 1995 [31]	Credit card usage	Intention, Extensive Margin, Intensive Margin	Field	246	ATE	Loss framing increased card usage.
Hastings et al., 2011 [34]	Investment choice	Intention	Field	8,614	ATE	Gain framing led to better fund choice. The effect was moderated by financial literacy and wealth.
Kim & McKinnon, 2020 [32]	Retirement saving	Intention	Lab	123 & 122	ATE	Loss framing led to earlier saving options when saving was framed as a process. Gain framing led to earlier saving options when saving was framed as an outcome.
Yoon & La Ferle, 2018 [97]	Retirement saving	Intention	Lab	188 & 180	ATE	Loss frames with self-orientation were more effective for lower collectivistic personalities. Loss frames with family-orientation were more effective for stronger collectivistic personalities.
**Our study**	**Retirement saving**	**Extensive Margin**, **Intensive Margin**	**Field**	**41,207**	CATE	**Large heterogeneity in effects when analyzing individual-level treatment effects that are otherwise masked by population-average estimates, with large potential for personalization.**

Experimental materials

All reminder messages were sent in the contract language (i.e., the language chosen at the beginning of the contract). Consequently, in addition to English, all major languages of Switzerland, including German, Italian, and French, were covered. In addition to the app illustrated in Figure 2, figures S1 and S2 show the German reminders for the loss condition and the gain condition, respectively.

[redacted]08.11.[redacted]

Lassen Sie sich Ihren Steuervorteil [redacted] nicht entgehen.

Sehr geehrte [redacted]

Sie haben sich im Rahmen Ihrer Altersvorsorge für eine 3a-Police [redacted] entschieden und zählen zu unseren sehr geschätzten Kunden.

Lassen Sie sich das gesamte Potenzial Ihrer Altersvorsorge nicht entgehen und zahlen Sie jetzt in Ihre Säule 3a bei der [redacted] ein, um Ihre finanziellen Spielräume im Alter beizubehalten. Vermeiden Sie den Verlust wertvoller Steuervorteile und reduzieren Sie Ihre Steuerlast. Einzahlungen in Ihre Säule 3a können Sie in vollem Umfang bei der Einkommensteuer geltend machen.

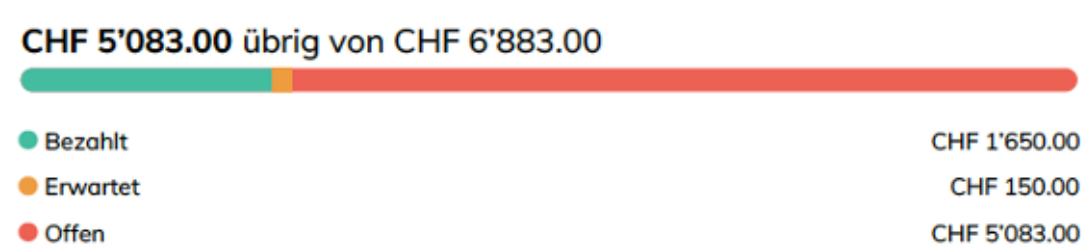

Um den Steuervorteil nicht zu verpassen, empfehlen wir Ihnen, noch in diesem Jahr eine Zuzahlung in Höhe von **CHF 5'083.00** in Ihre Police zu tätigen.

Als Erwerbstätige mit Pensionskasse können Sie im [redacted] **maximal CHF 6'883.00** in Ihre Vorsorge einzahlen.

Der einfachste Weg, in Ihre 3a-Police einzuzahlen, ist die Verwendung der [redacted] Hier finden Sie immer aktuelle Informationen zu Ihrer 3a-Police und können Ihren Fortschritt auch während des Jahres verfolgen. Alternativ können Sie für Ihre Zuzahlung auch den beiliegenden Einzahlungsschein verwenden.

Damit Ihre Zuzahlung für das Jahr [redacted] steuerlich anerkannt werden kann, muss diese bis zum **26. Dezember** [redacted] (Valuta [redacted] bei uns eintreffen.

Wenn Sie mehrere 3a-Verträge haben, gilt die gesetzliche Höchstgrenze nicht pro Vertrag, sondern für alle Verträge zusammen. Insgesamt dürfen alle Zahlungen (z.B. Prämien, Zuzahlungen) die Höchstgrenze nicht überschreiten.

Ihr Versicherungsberater und das Team [redacted] stehen Ihnen jederzeit gerne zur Verfügung.

Freundliche Grüsse

[redacted]

Figure S1: **Reminder loss framing:** German version of the loss-framed message.

[redacted] 08.11. [redacted]

Sichern Sie sich Ihren Steuervorteil [redacted]

Sehr geehrte [redacted]

Sie haben sich im Rahmen Ihrer Altersvorsorge für eine 3a-Police [redacted] entschieden und zählen zu unseren sehr geschätzten Kunden.

Nutzen Sie das gesamte Potenzial Ihrer Altersvorsorge und zahlen Sie jetzt in Ihre Säule 3a [redacted] ein, um Ihren finanziellen Spielraum im Alter zu maximieren. Profitieren Sie zusätzlich von Steuervorteilen und reduzieren Sie Ihre Steuerlast. Einzahlungen in Ihre Säule 3a können Sie in vollem Umfang bei der Einkommensteuer geltend machen.

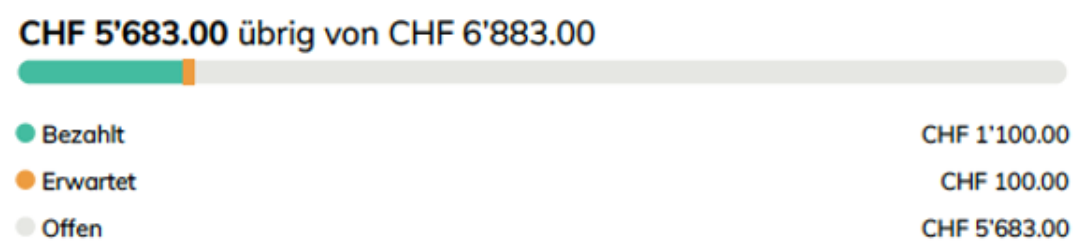

Um die gesetzliche Höchstgrenze voll auszuschöpfen, empfehlen wir Ihnen, noch in diesem Jahr eine Zuzahlung in Höhe von **CHF 5'683.00** in Ihre Police zu tätigen.

Als Erwerbstätige mit Pensionskasse können Sie im [redacted] **maximal CHF 6'883.00** in Ihre Vorsorge einzahlen.

Der einfachste Weg, in Ihre 3a-Police einzuzahlen, ist die Verwendung der [redacted] Hier finden Sie immer aktuelle Informationen zu Ihrer 3a-Police und können Ihren Fortschritt auch während des Jahres verfolgen. Alternativ können Sie für Ihre Zuzahlung auch den beiliegenden Einzahlungsschein verwenden.

Damit Ihre Zuzahlung für das Jahr [redacted] steuerlich anerkannt werden kann, muss diese bis zum **26. Dezember** [redacted] (Valuta [redacted] bei uns eintreffen.

Wenn Sie mehrere 3a-Verträge haben, gilt die gesetzliche Höchstgrenze nicht pro Vertrag, sondern für alle Verträge zusammen. Insgesamt dürfen alle Zahlungen (z.B. Prämien, Zuzahlungen) die Höchstgrenze nicht überschreiten.

Ihr Versicherungsberater und das Team [redacted] stehen Ihnen jederzeit gerne zur Verfügung.

Freundliche Grüsse
[redacted]

Figure S2: **Reminder gain framing:** German version of the gain-framed message.

Variable descriptions

Table S2: Variables.

- `Conversion`: Binary information on whether a customer made an additional payment ($Y = 1$) or not ($Y = 0$) between the intervention and the end of the observation period—the extensive margin.
- `Savings Amount`: Numeric value in euros of the paid payment during the observation period—the intensive margin.
- `Gender`: Binary information on gender for females ($X = 1$) and males ($X = 0$).
- `Age`: Numeric value of the age of a customer at the time of the intervention in years.
- `Contract Valuation`: Contract value in million euros of a customer measured by the sum of all committed payments until the end of the contract at the time of the intervention.
- `Number of Historical Payments`: Integer of the number of previous one-time payments.
- `Employment Status`: Boolean information whether the participant is part of a company pension plan and therefore employed with a company (then the value is true) or self-employed (then the value is false).
- `Information Skills`: Numeric value measuring skills for collecting, storing, monitoring, and using information [88].
- `Computer Skills`: Numeric value measuring skills for using computers and other digital tools [88].
- `Management Skills`: Numeric value measuring skills for managing people, activities, and resources [88].
- `Communication, Collaboration, and Creativity Skills`: Numeric value measuring skills for communicating, collaborating, and creating [88].
- `Assisting and Caring Skills`: Numeric value measuring skills for providing assistance, care, and service [88].
- `Construction Skills`: Numeric value measuring skills for building, repairing, and installing structures [88].
- `Machinery and Equipment Skills`: Numeric value measuring skills for controlling and operating vehicles and machinery [88].
- `Handling and Moving Skills`: Numeric value measuring skills for sorting, arranging, moving, and transforming goods [88].

Summary statistics

Table S3: **Summary statistics for Field Experiment 1** (N = 22,919; gain framing N = 7,670, loss framing N = 15,249). The difference between the experimental groups for the covariate 'Computer Skills' (F = 6.218, p = 0.013) is likely due to the large sample size of our study; the corresponding standardized mean difference is with 0.034 well below the threshold of 0.1, which indicates good covariate balance between experimental conditions [87].

**Variable**	**Condition: Gain framing**		**Condition: Loss framing**		***F*-Statistic**
	**Mean**	**SD**	**Mean**	**SD**	
Gender: Female	0.41	0.49	0.41	0.49	0.007
Age	36	9.5	36	9.8	1.571
Employment Status: Employed	0.85	0.36	0.85	0.36	0.009
Contract Valuation	0.089	0.048	0.090	0.050	2.109
Number of Historical Payments	0.10	0.51	0.094	0.46	1.453
Information Skills	0.17	0.11	0.17	0.11	1.125
Computer Skills	0.035	0.061	0.033	0.054	6.218*
Management Skills	0.12	0.11	0.12	0.11	1.394
Communic., Collab., Creativity	0.24	0.16	0.24	0.16	0.144
Assisting and Caring Skills	0.14	0.12	0.14	0.12	0.000
Construction Skills	0.037	0.078	0.038	0.077	0.450
Machinery and Equipment Skills	0.055	0.089	0.058	0.091	2.870
Handling and Moving Skills	0.095	0.13	0.094	0.13	0.084

SD: standard deviation; Statistical significance: * $p < 0.05$; ** $p < 0.01$; *** $p < 0.001$

Table S4: **Summary statistics for Field Experiment 2** (N = 18,288; gain framing N = 9,087, loss framing N = 9,201). No covariate differs significantly between the experimental conditions at the 5% level. All CATE, ATE, RATE, and policy estimates reported in this paper are evaluated on this experiment.

**Variable**	**Condition: Gain framing**		**Condition: Loss framing**		***F*-Statistic**
	**Mean**	**SD**	**Mean**	**SD**	
Gender: Female	0.41	0.49	0.41	0.49	0.338
Age	36	9.5	36	9.9	1.755
Employment Status: Employed	0.84	0.37	0.84	0.37	0.035
Contract Valuation	0.088	0.048	0.088	0.048	0.005
Number of Historical Payments	0.12	0.55	0.12	0.52	0.008
Information Skills	0.17	0.11	0.17	0.11	0.921
Computer Skills	0.034	0.058	0.032	0.054	3.722
Management Skills	0.12	0.11	0.12	0.11	0.537
Communic., Collab., Creativity	0.24	0.16	0.24	0.16	0.709
Assisting and Caring Skills	0.14	0.12	0.14	0.12	0.006
Construction Skills	0.038	0.078	0.038	0.078	0.496
Machinery and Equipment Skills	0.056	0.089	0.056	0.089	0.321
Handling and Moving Skills	0.094	0.13	0.094	0.13	0.065

SD: standard deviation; Statistical significance: * $p < 0.05$; ** $p < 0.01$; *** $p < 0.001$

Evaluation of personalized targeting policy

We employ the Targeting Operator Characteristic (TOC) curve to diagnose the model's treatment prioritization capability on the validation set.

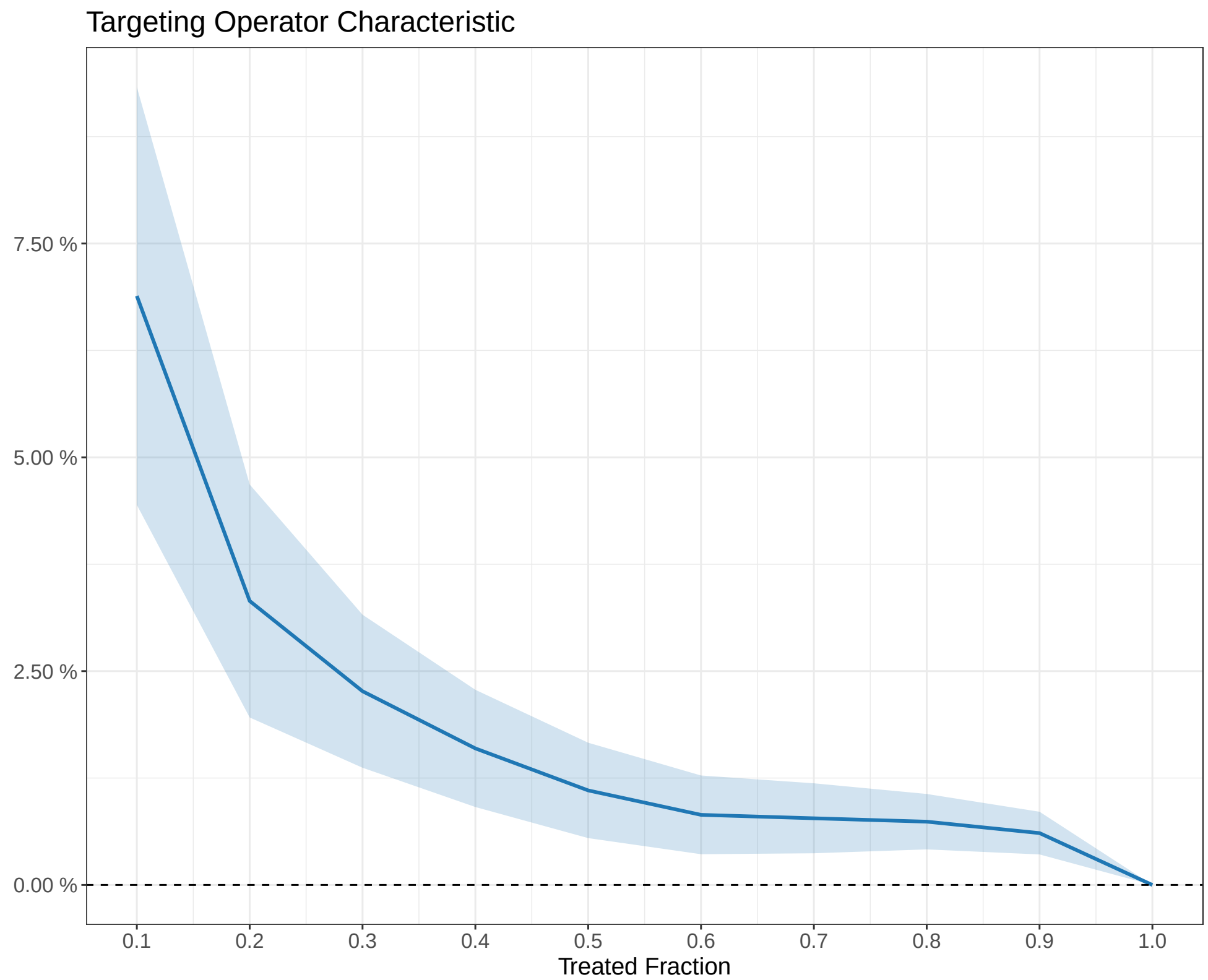

Figure S3: **Targeting Operator Characteristic (TOC)** for the CATE-based policy with conversion as the dependent variable. The area under the TOC, or RATE, is 2.59 (95% CI: 1.72, 3.46).

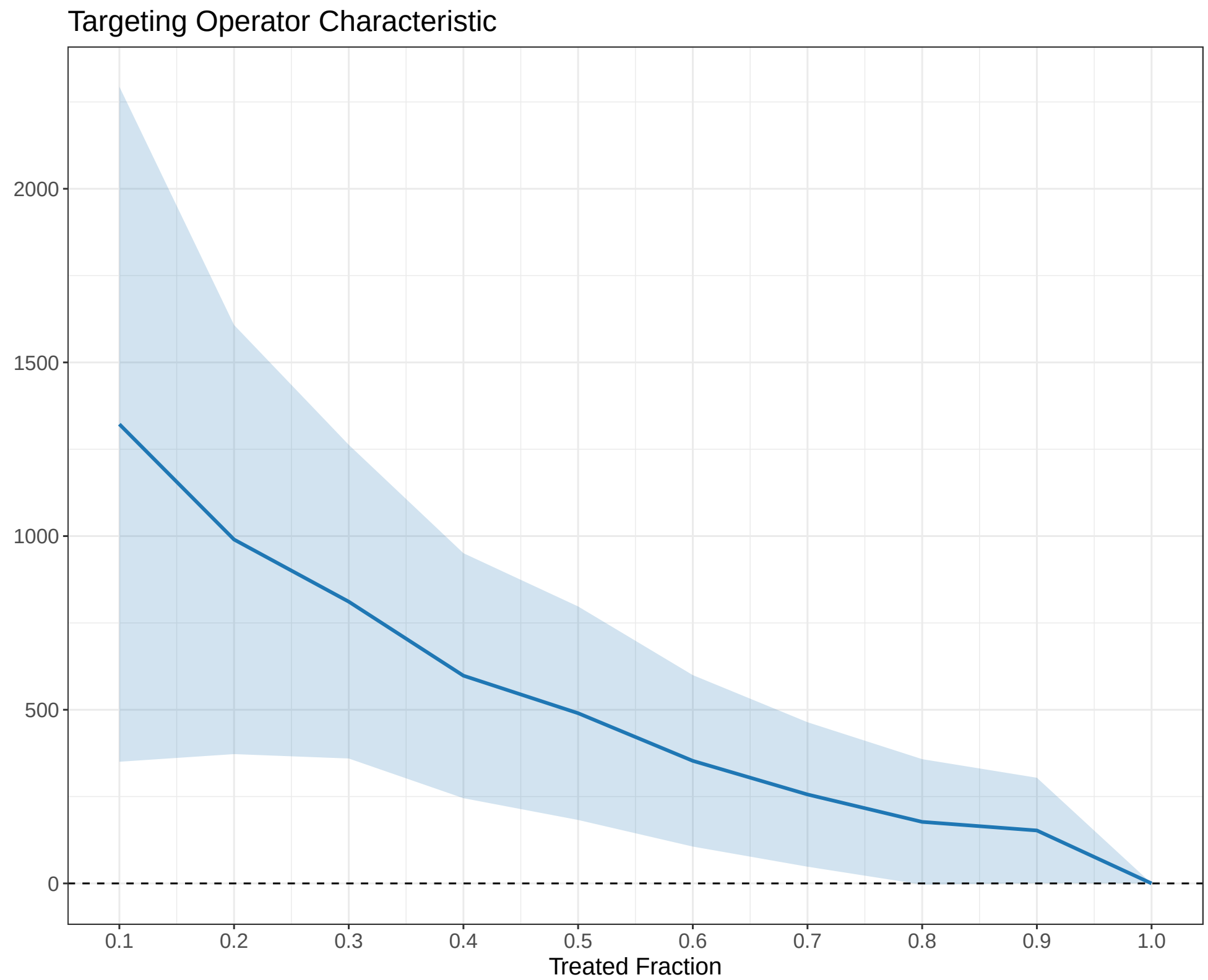

Figure S4: **Targeting Operator Characteristic (TOC)** for the CATE-based policy with saving amount as the dependent variable. The area under the TOC, or RATE, is €665 (95% CI: €306, €1,024).

Additional analysis of potential effect modifiers

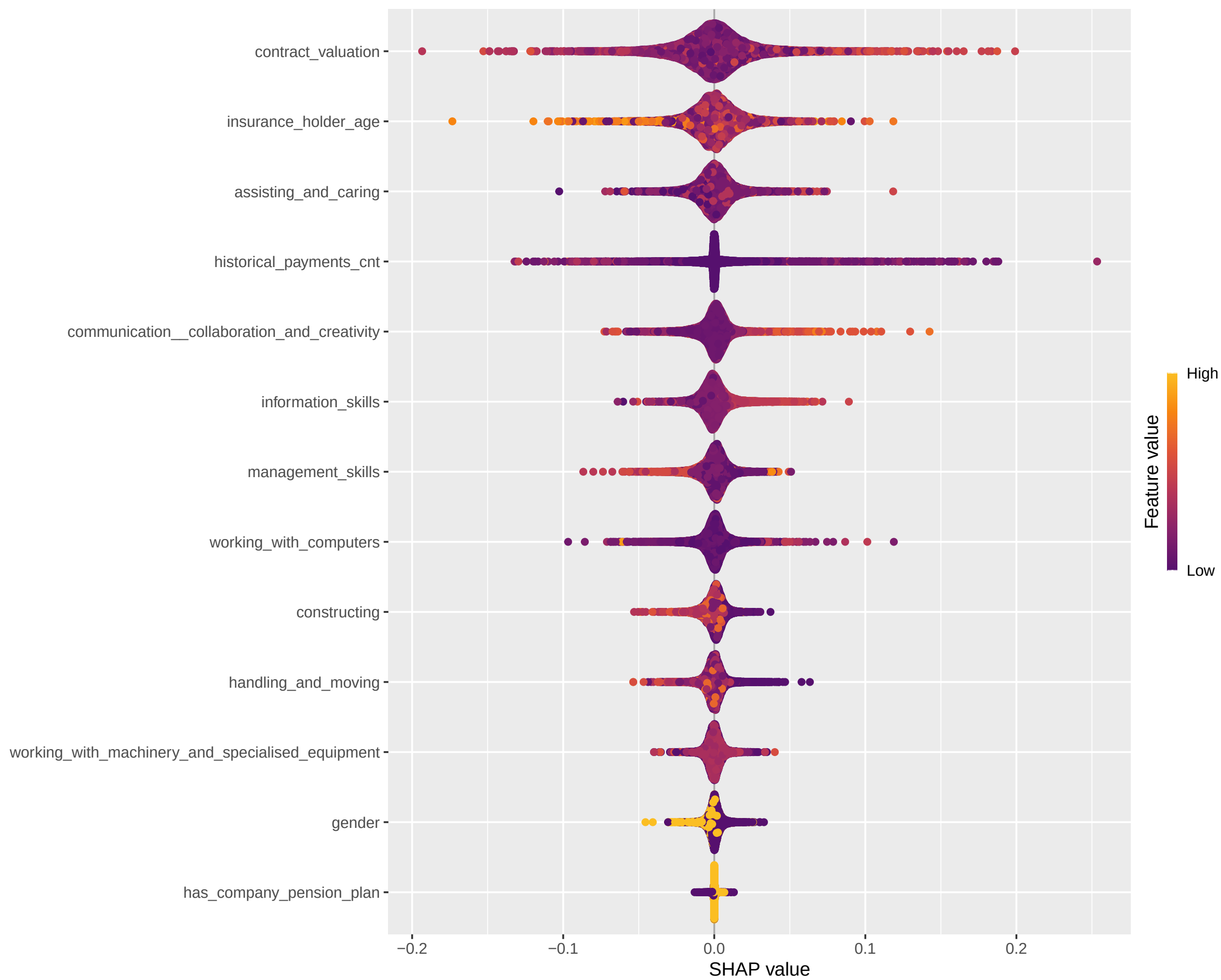

Figure S5: **SHAP summary plot for conversion.** The plot shows global feature importance (in descending order on the y-axis) as well as direction and magnitude of relationships between feature values and SHAP values (x-axis and hue).

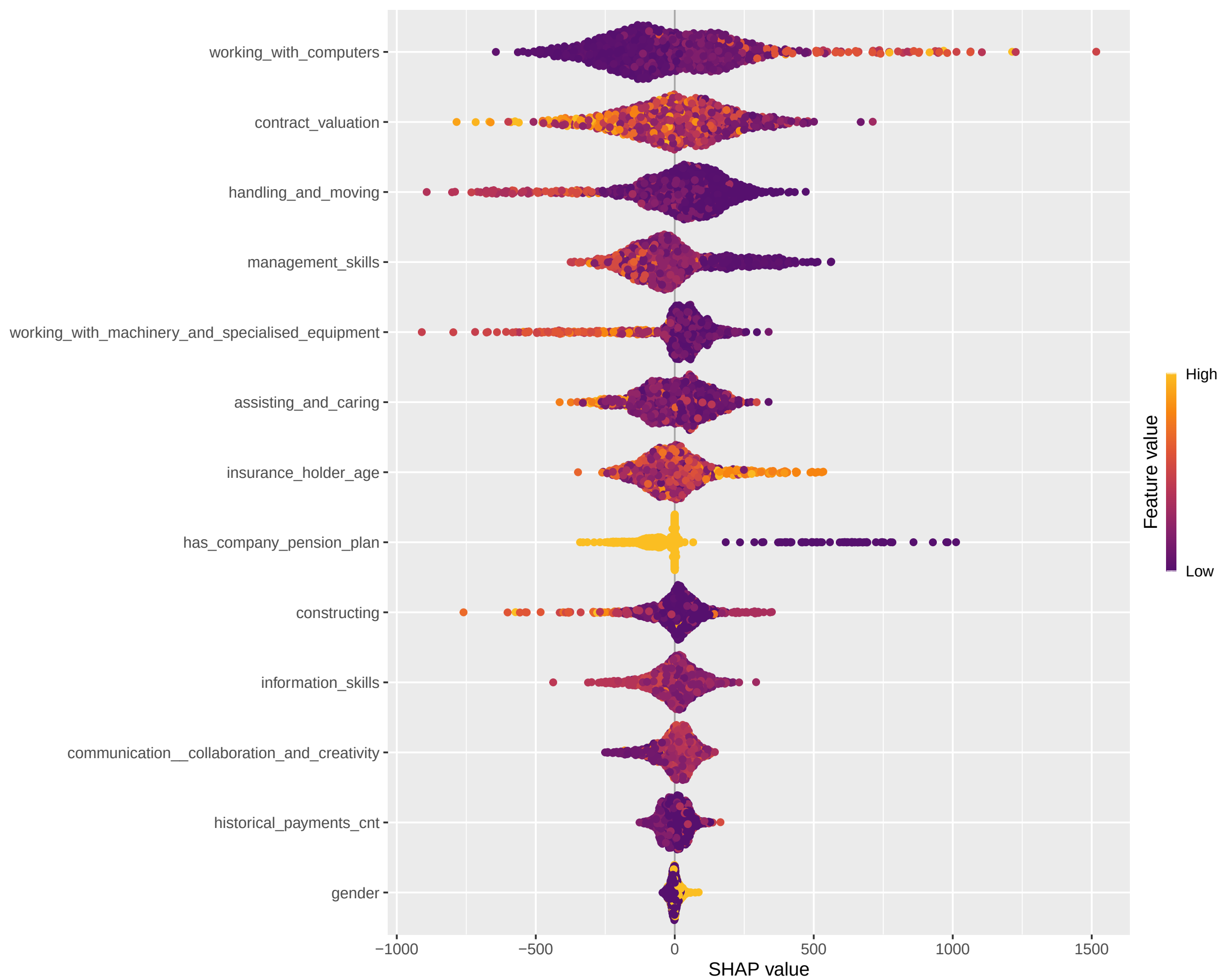

Figure S6: **SHAP summary plot for saving amount.** The plot shows global feature importance (in descending order on the y-axis) as well as direction and magnitude of relationships between feature values and SHAP values (x-axis and hue).

Economic impact analysis with alternative targeting rules

The following figures visualize the effectiveness of simple rule-based policies inspired by existing theory and related work.

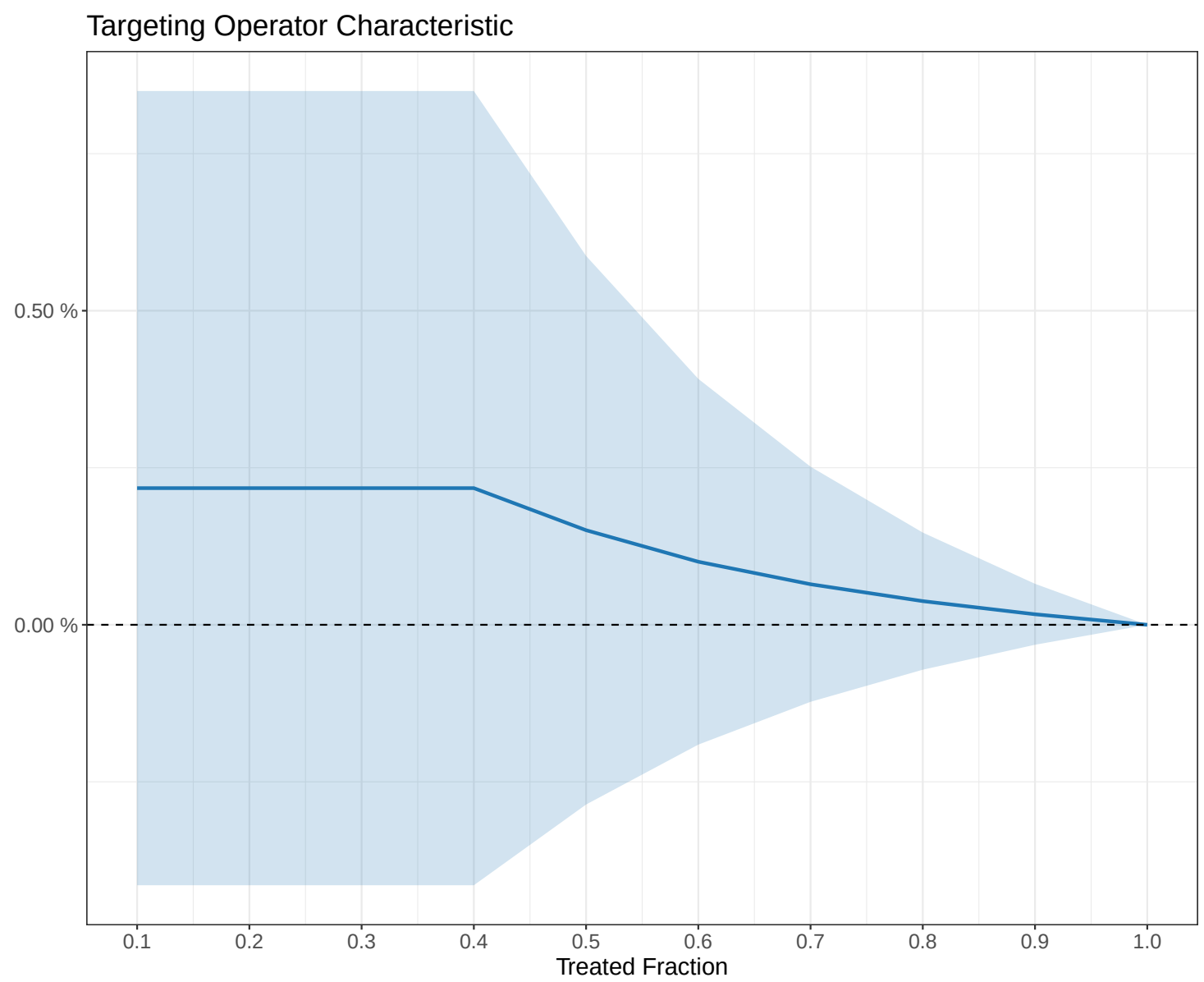

Figure S7: **Targeting Operator Characteristic (TOC)** for a simple rule-based policy based on **gender** and with **conversion** as the dependent variable. The area under the TOC, or RATE, is 0.13 (95% CI: -0.27, 0.54), indicating an absence of effect heterogeneity with regard to gender.

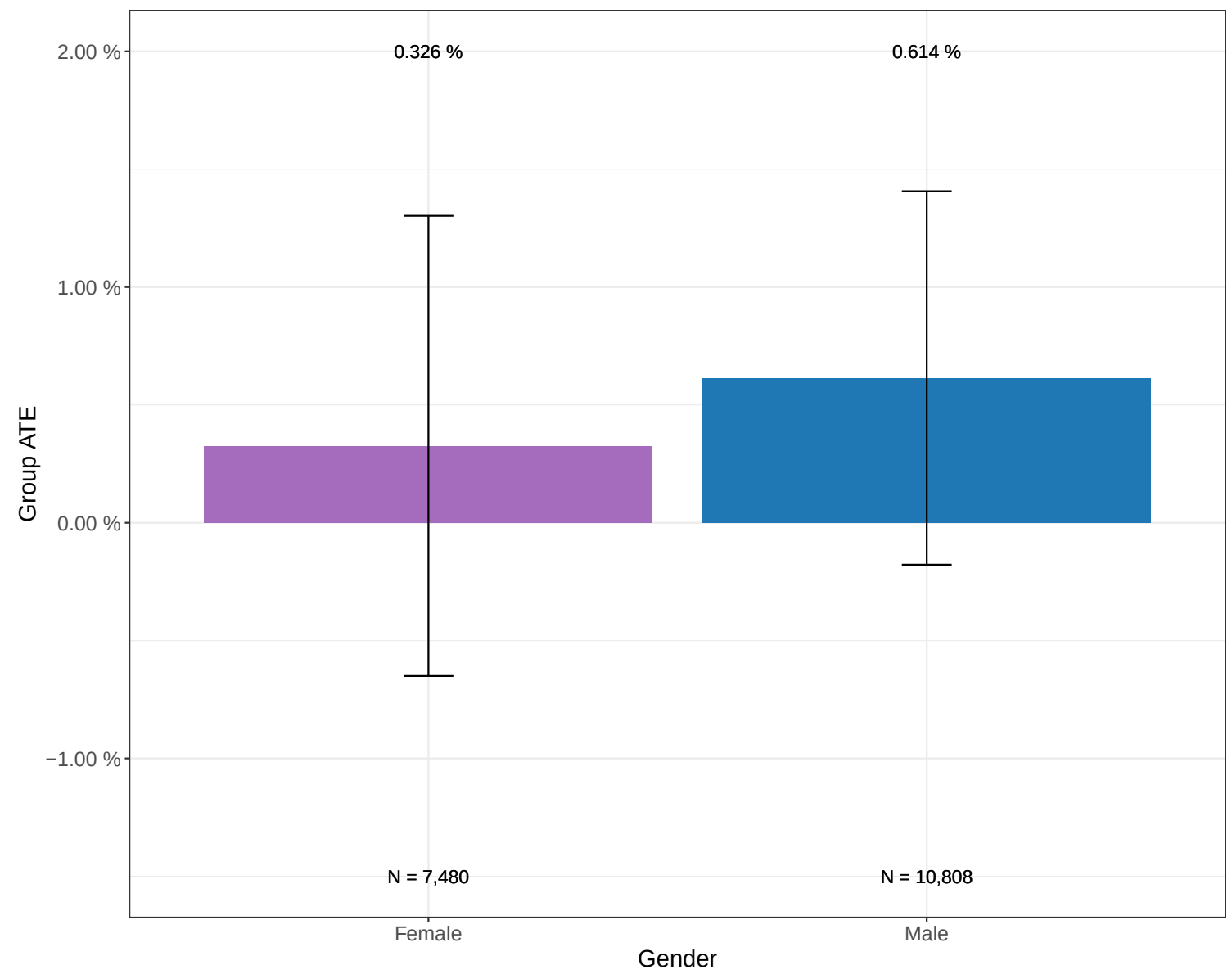

Figure S8: Group ATE of sending a loss-framed message on **conversion** probability aggregated by a **targeting rule based on gender**. The results suggest that personalization by gender alone is ineffective.

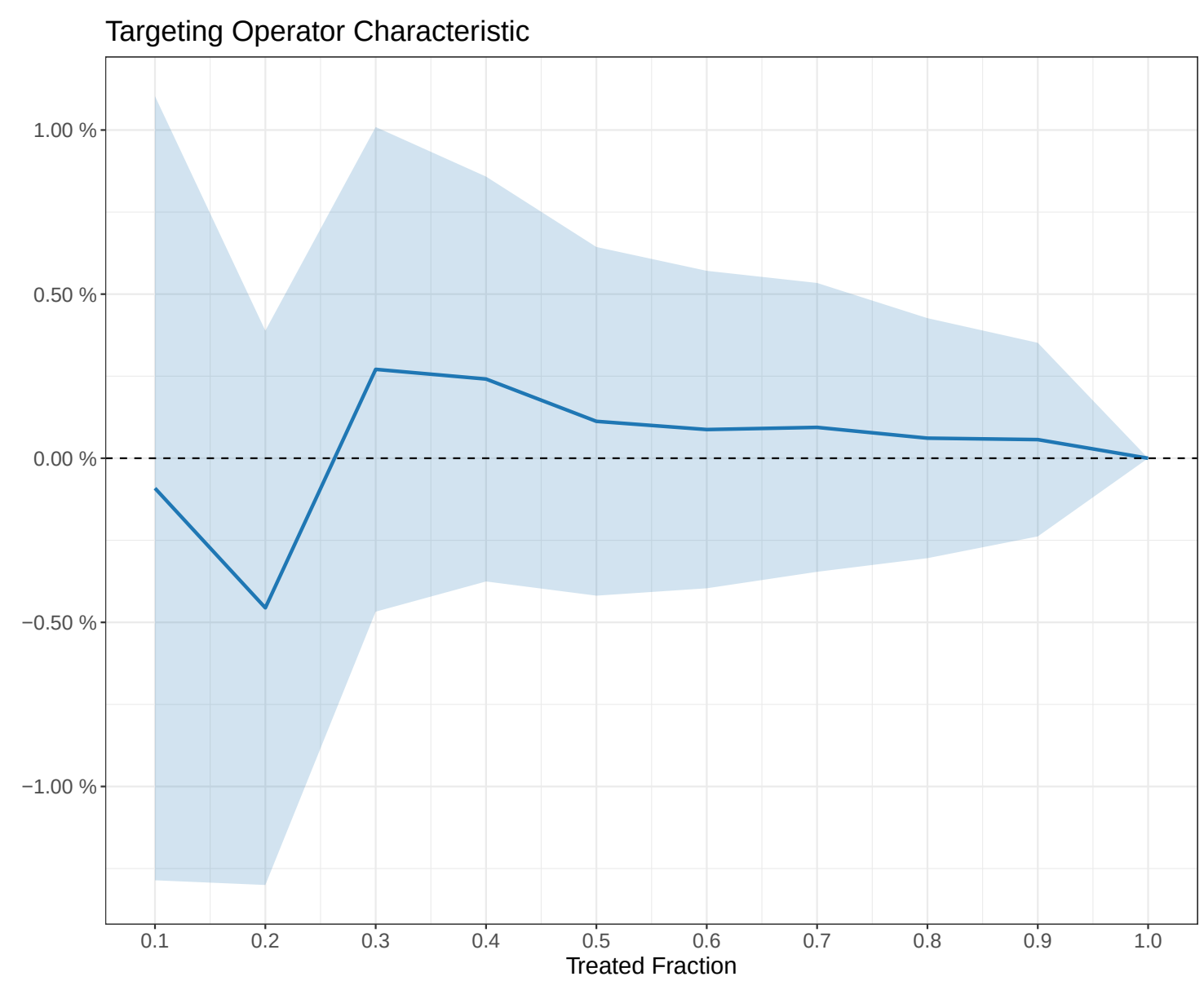

Figure S9: **Targeting Operator Characteristic (TOC)** for a simple rule-based policy based on **contract valuation**—as a proxy for wealth—and with **conversion** as the dependent variable. The area under the TOC, or RATE, is -0.14 (95% CI: -0.66, 0.38), indicating an absence of effect heterogeneity with regard to wealth.

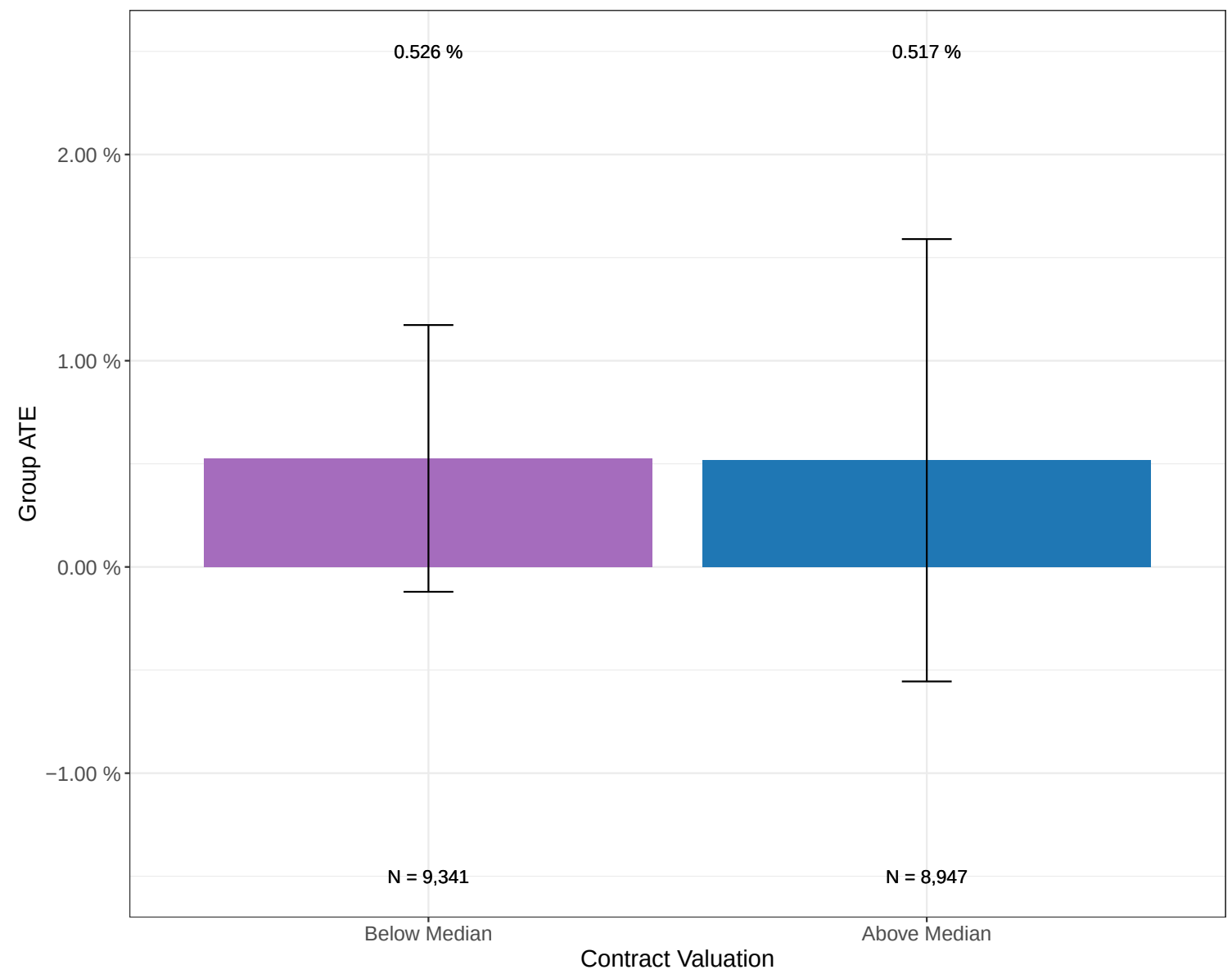

Figure S10: Group ATE of sending a loss-framed message on **conversion** probability aggregated by a **targeting rule based on contract valuation** as a proxy for wealth. The results suggest that personalization by wealth alone is ineffective.

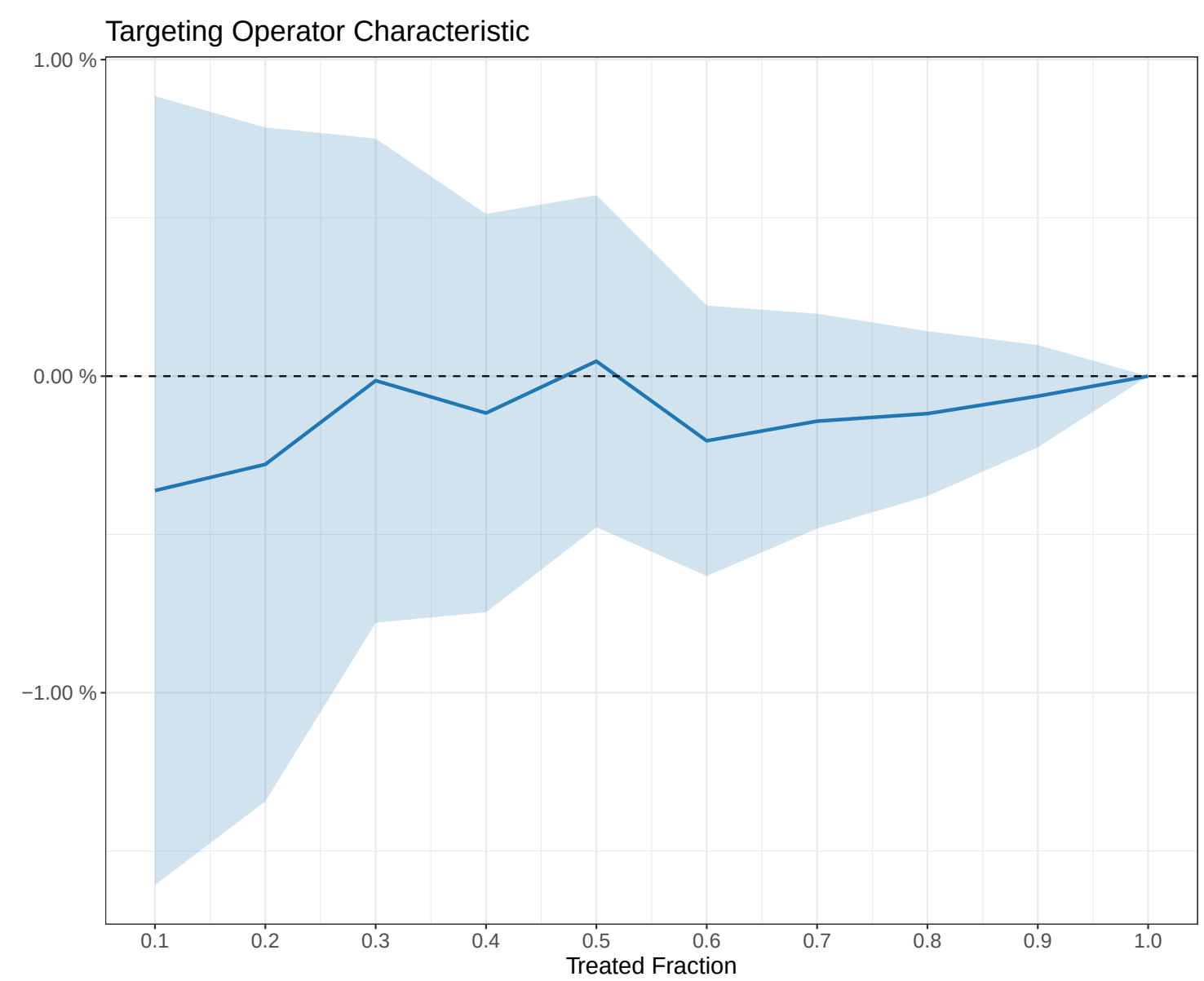

Figure S11: **Targeting Operator Characteristic (TOC)** for a simple rule-based policy based on **management skills—**as a proxy for financial literacy—and with **conversion** as the dependent variable. The area under the TOC, or RATE, is -0.15 (95% CI: -0.61, 0.30), indicating an absence of effect heterogeneity with regard to financial literacy.

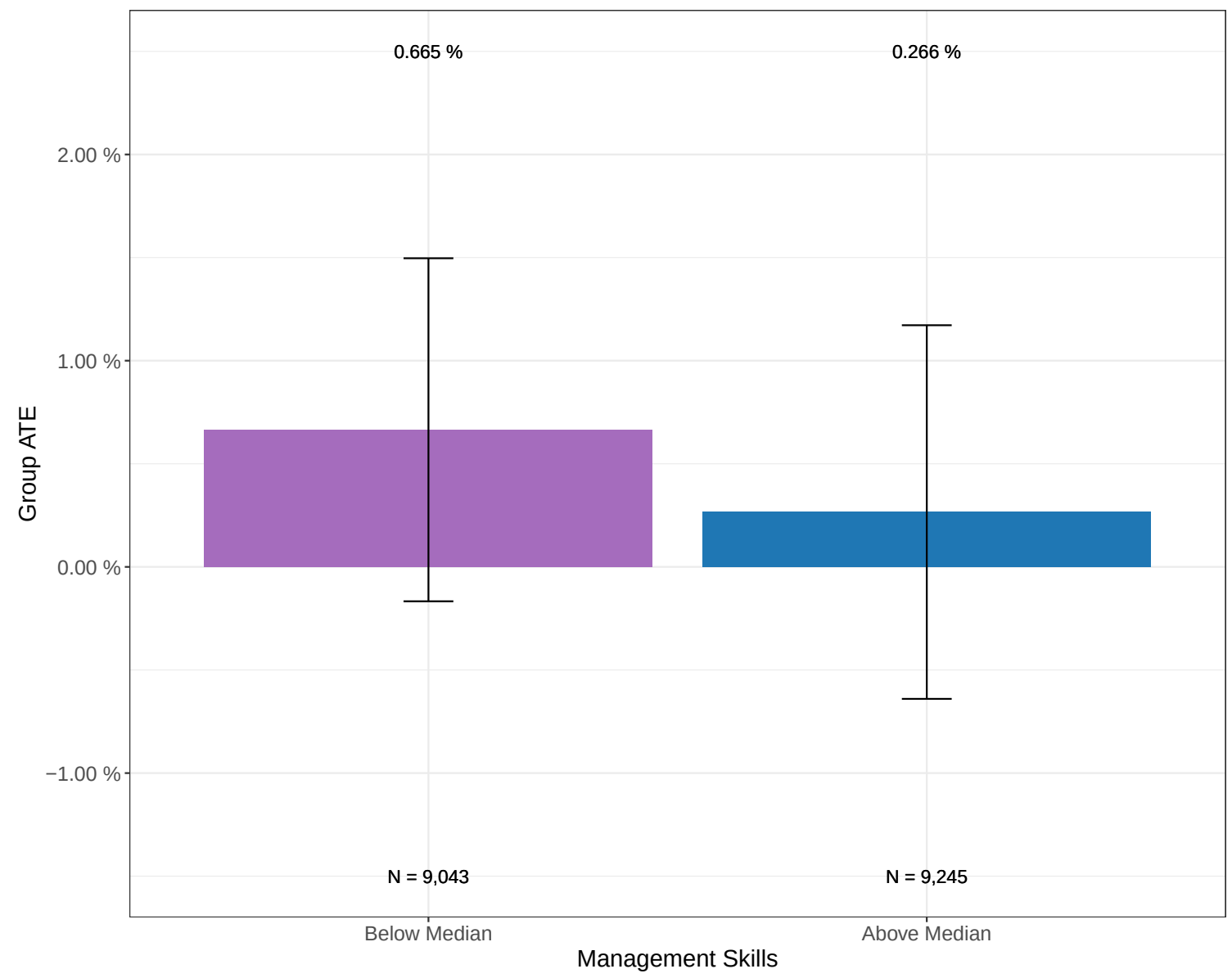

Figure S12: Group ATE of sending a loss-framed message on **conversion** probability aggregated by a **targeting rule based on management skills** as a proxy for financial literacy. The results suggest that personalization by financial literacy alone is ineffective.

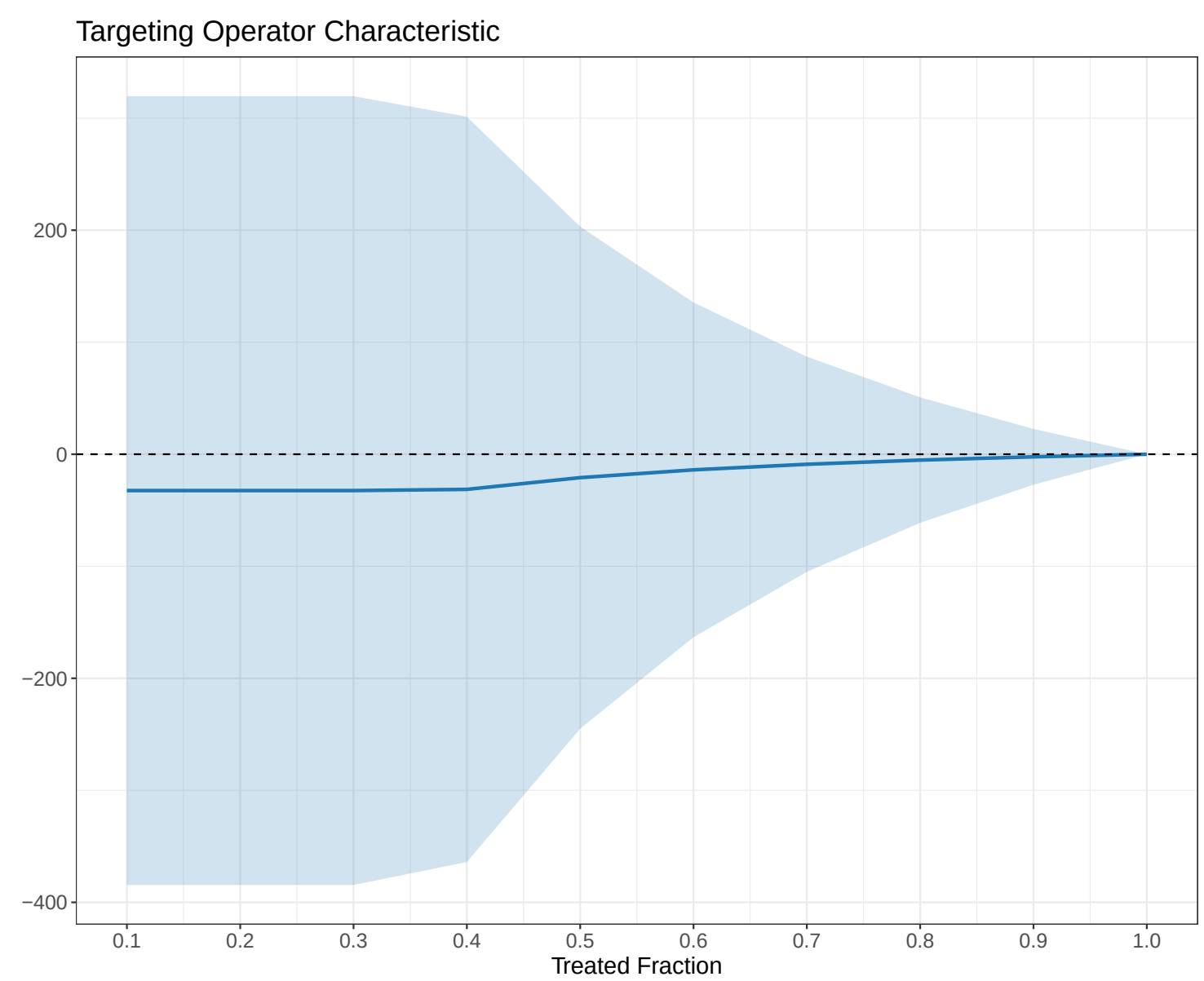

Figure S13: **Targeting Operator Characteristic (TOC)** for a simple rule-based policy based on **gender** and with **saving amount** as the dependent variable. The area under the TOC, or RATE, is €-19.57 (95% CI: -230.41, 191.27), indicating an absence of effect heterogeneity with regard to gender.

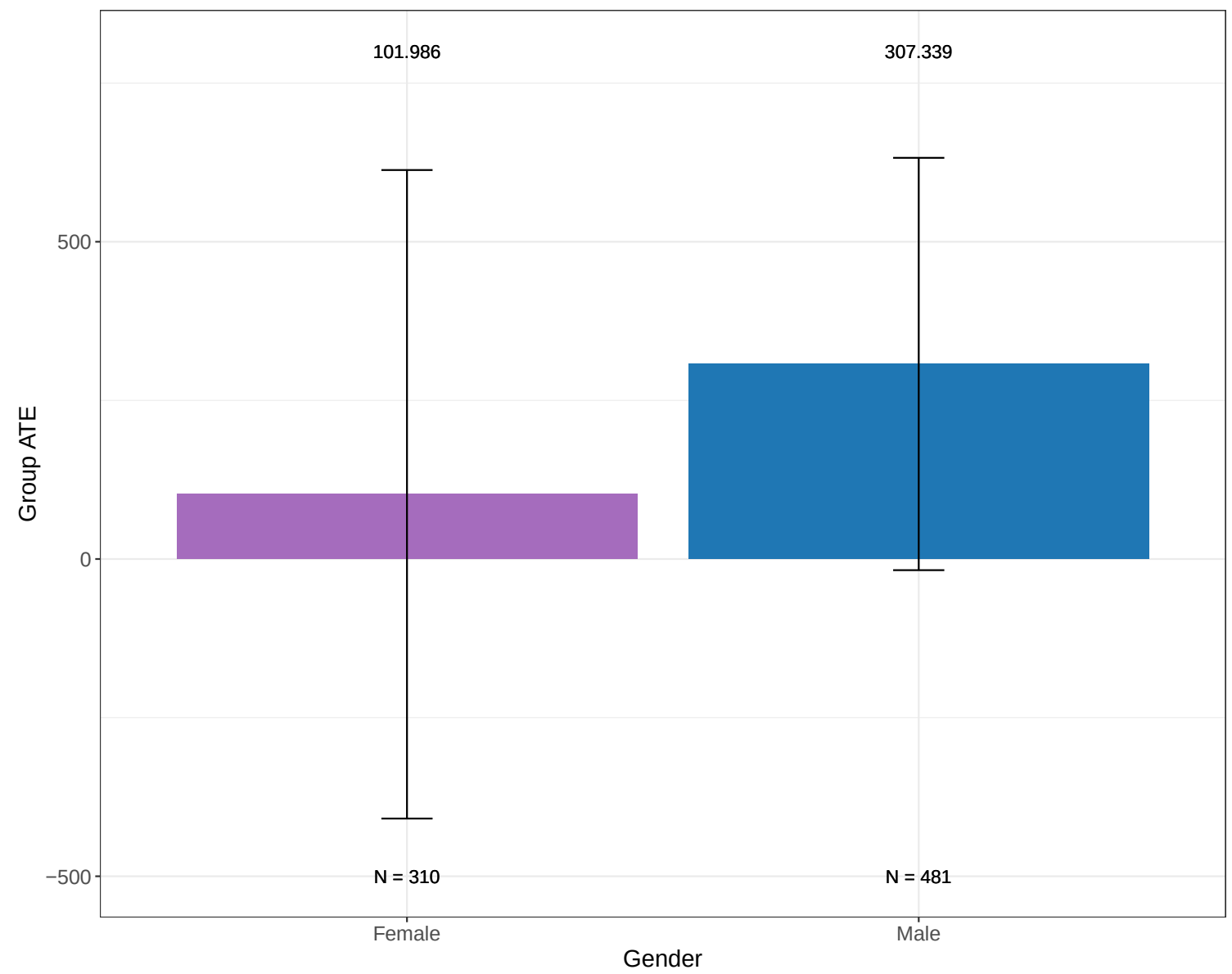

Figure S14: Group ATE of sending a loss-framed message on **saving amount** aggregated by a **targeting rule based on gender**. The results suggest that personalization by gender alone is ineffective.

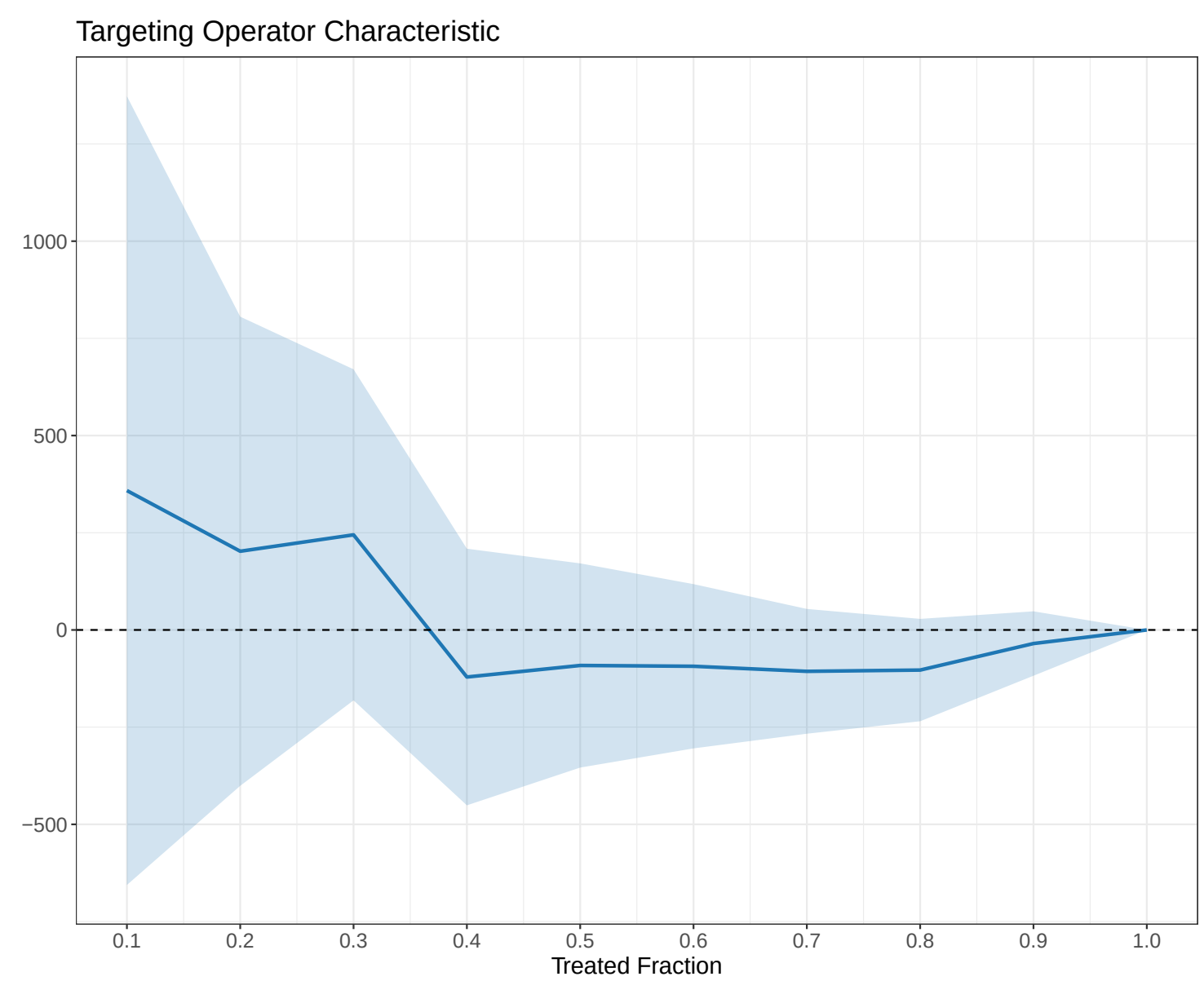

Figure S15: **Targeting Operator Characteristic (TOC)** for a simple rule-based policy based on **contract valuation**—as a proxy for wealth—and with **saving amount** as the dependent variable. The area under the TOC, or RATE, is €38.85 (95% CI: -247.87, 325.56), indicating an absence of effect heterogeneity with regard to wealth.

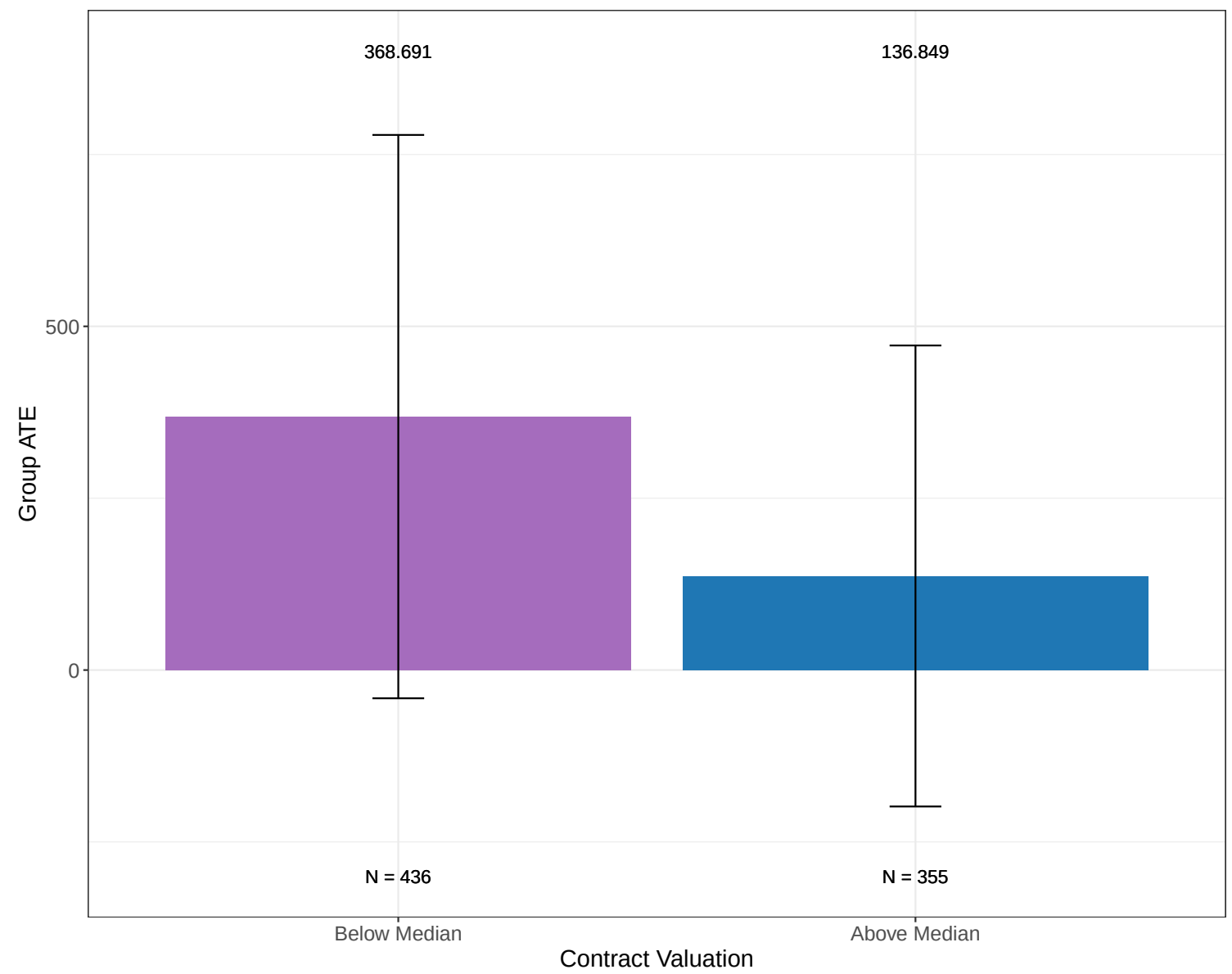

Figure S16: Group ATE of sending a loss-framed message on **saving amount** aggregated by a **targeting rule based on contract valuation** as a proxy for wealth. The results suggest that personalization by wealth alone is ineffective.

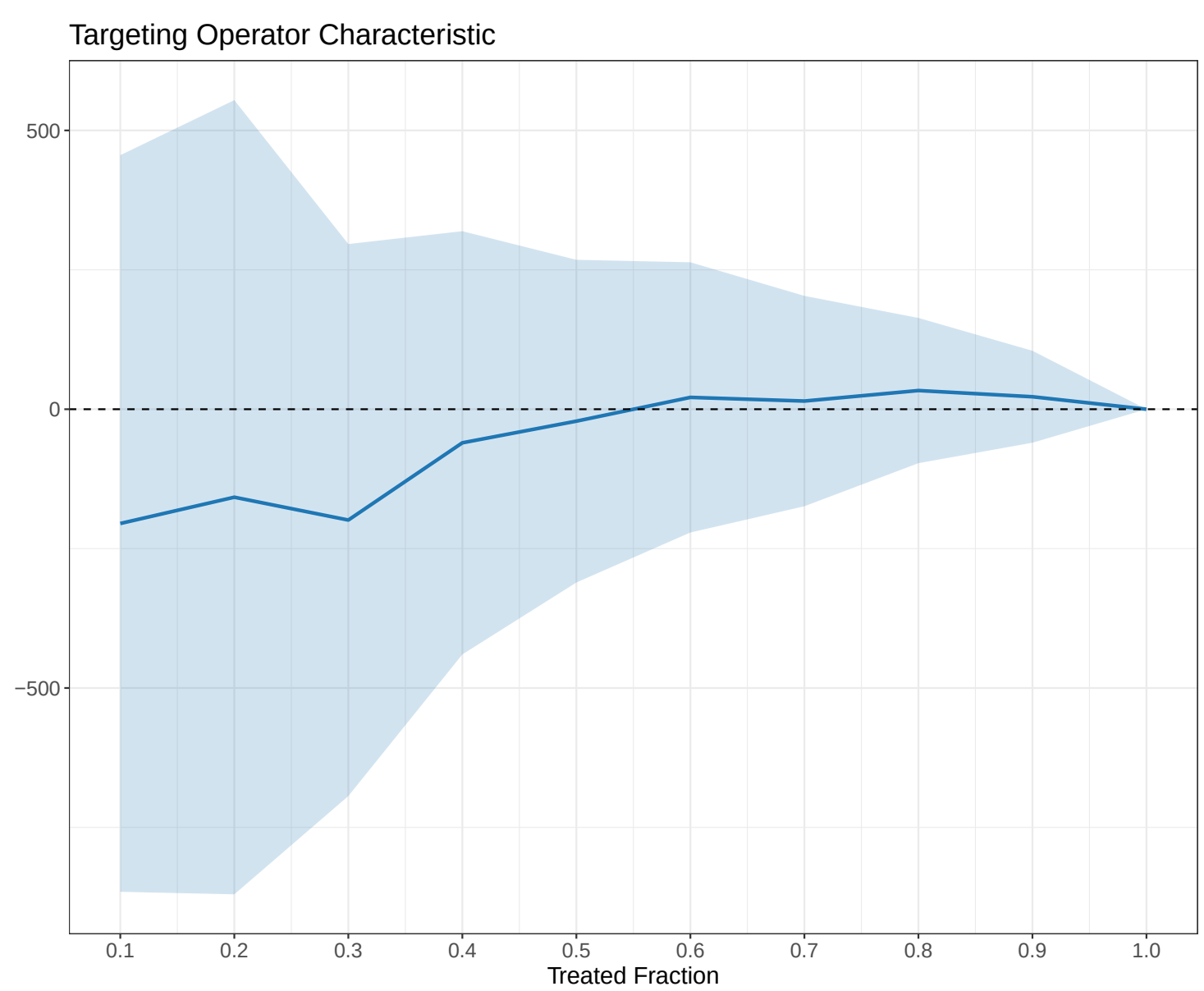

Figure S17: **Targeting Operator Characteristic (TOC)** for a simple rule-based policy based on **management skills**—as a proxy for financial literacy—and with **saving amount** as the dependent variable. The area under the TOC, or RATE, is €-79.86 (95% CI: -339.11, 179.39), indicating an absence of effect heterogeneity with regard to financial literacy.

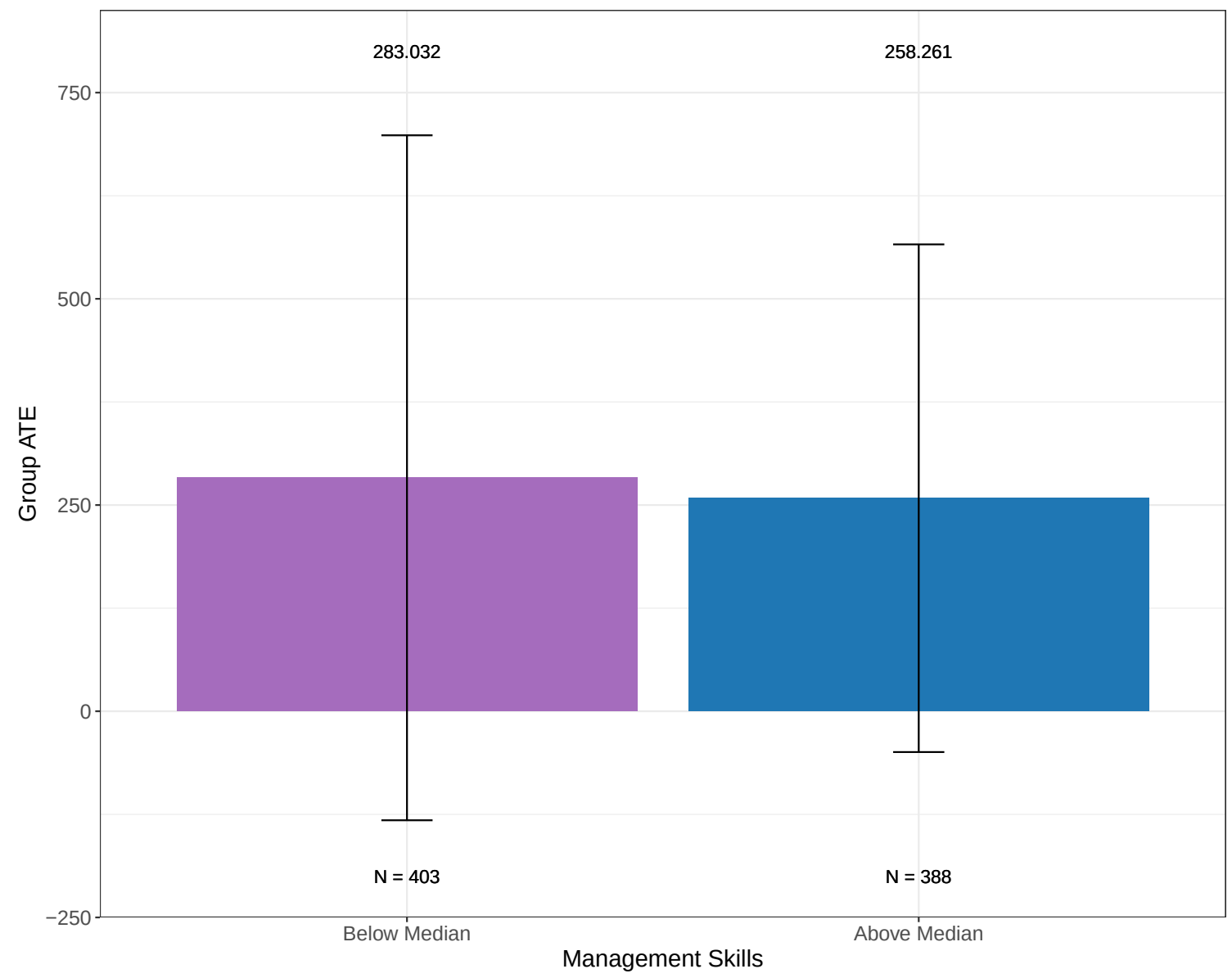

Figure S18: Group ATE of sending a loss-framed message on **saving amount** aggregated by a **targeting rule based on management skills** as a proxy for financial literacy. The results suggest that personalization by financial literacy alone is ineffective.

Additional robustness checks for ATE estimation

In addition to using an AIPW estimator for estimating the ATE, we also applied more traditional statistical models. First, we estimated the ATE of loss- versus gain-framed messages using standard probit and OLS specifications for the extensive and intensive margins. Second, we addressed potential sample-selection bias in the intensive margin by estimating a Heckman two-step selection model [99]. Across all specifications, experiments, and outcome measures, the results remain unchanged: loss-framed and gain-framed messages do not differ in their average effectiveness.

The sample used to estimate the intensive margin is a nonrandomly selected sample, as it depends on the sample of the first step. Therefore, to prevent a sample-selection bias in the second step, we estimated a Heckman two-step correction model [99]. Following Bekkers and Crutzen [100], we use the number of historical payments from earlier years as the additional independent variable in the selection model. The selection equation is:

$$\mathrm{Y}_2 = \alpha Z + \delta \tag{S1}$$

The estimates are carried over through the inverse Mills ratio $\lambda(T - \alpha Z)$ [101, 99]:

$$\mathrm{Y}_1 = B_0 + B_1 X + \sigma \rho_{\varepsilon\delta} \lambda(T - \alpha Z) + \sigma' \varepsilon' \tag{S2}$$

To estimate the effect on the *extensive margin* we ran a Probit model:

$$P(y = 1 \mid X) = \Phi(X\beta) \tag{S3}$$

To analyze the effect on the *intensive margin* an OLS linear regression model was estimated:

$$y = X\beta + \varepsilon. \tag{S4}$$

Table S5: **Estimated average treatment effects (ATE)** of loss-framed messages (versus gain-framed messages) on the extensive and intensive margin for Experiment 1.

Model	Probit	OLS	Selection Model	
Dependent Variable:	Extensive	Intensive	Extensive	Intensive
	Converted	Saving Amount (log)	Converted	Saving Amount (log)
	(1)	(2)	(3)	(4)
**Condition and Instrumental Variable**				
Constant	-1.637***	6.351***	-1.768***	6.958***
Received Message: Loss	0.018	0.107	0.028	0.106
Historical Payments			0.566***	
Observations	22,919	1,195	22,919	22,919
R^2		0.001		
Adjusted R^2		0.0001		
Log Likelihood	-4,692.91			
Akaike Inf. Crit.	9,389.82			
ρ			-0.192	-0.192
Inverse Mills Ratio			-0.327***	-0.327***
Residual Std. Error		1.685		
F-Statistic		1.060		

Statistical significance markers: * 0.05, ** 0.01, *** 0.001.

Table S6: **Estimated average treatment effects (ATE)** of loss-framed messages (versus gain-framed messages) on the extensive and intensive margin for Experiment 2.

Model	Probit	OLS	Selection Model	
Dependent Variable:	Extensive	Intensive	Extensive	Intensive
	Converted	Saving Amount (log)	Converted	Saving Amount (log)
	(1)	(2)	(3)	(4)
**Condition and Instrumental Variable**				
Constant	-1.737***	6.701***	-1.932***	6.515***
Received Message: Loss	0.045	0.101	0.050	0.104
Historical Payments			0.634***	
Observations	18,288	791	18,288	18,288
R^2		0.001		
Adjusted R^2		-0.0003		
Log Likelihood	-3,256.98			
Akaike Inf. Crit.	6,517.96			
ρ			0.062	0.062
Inverse Mills Ratio			0.103	0.103
Residual Std. Error		1.650		
F-Statistic		0.745		

Statistical significance markers: * 0.05, ** 0.01, *** 0.001.

Additional robustness checks for CATE estimation

The estimated results outlined in Section Heterogeneous effects on the intensive margin (saving amount) are based on the subset of converted users. As an alternative approach and robustness check, the estimated ATE and CATEs presented in Figure S19 are based on a zero-imputed dataset. Accordingly, for customers that did not decide to make an additional payment, the invested amount is imputed with zero. As a result, the number of observations is larger (i.e., the same as for the estimated results of the extensive margin) and the absolute effect sizes smaller. Still, the directional

results are fully compatible with the analysis in Section Heterogeneous effects on the intensive margin (saving amount): the ATE of loss-framed messages on savings amount is not statistically significant, while the CATE distribution reveals substantial heterogeneity in both directions.

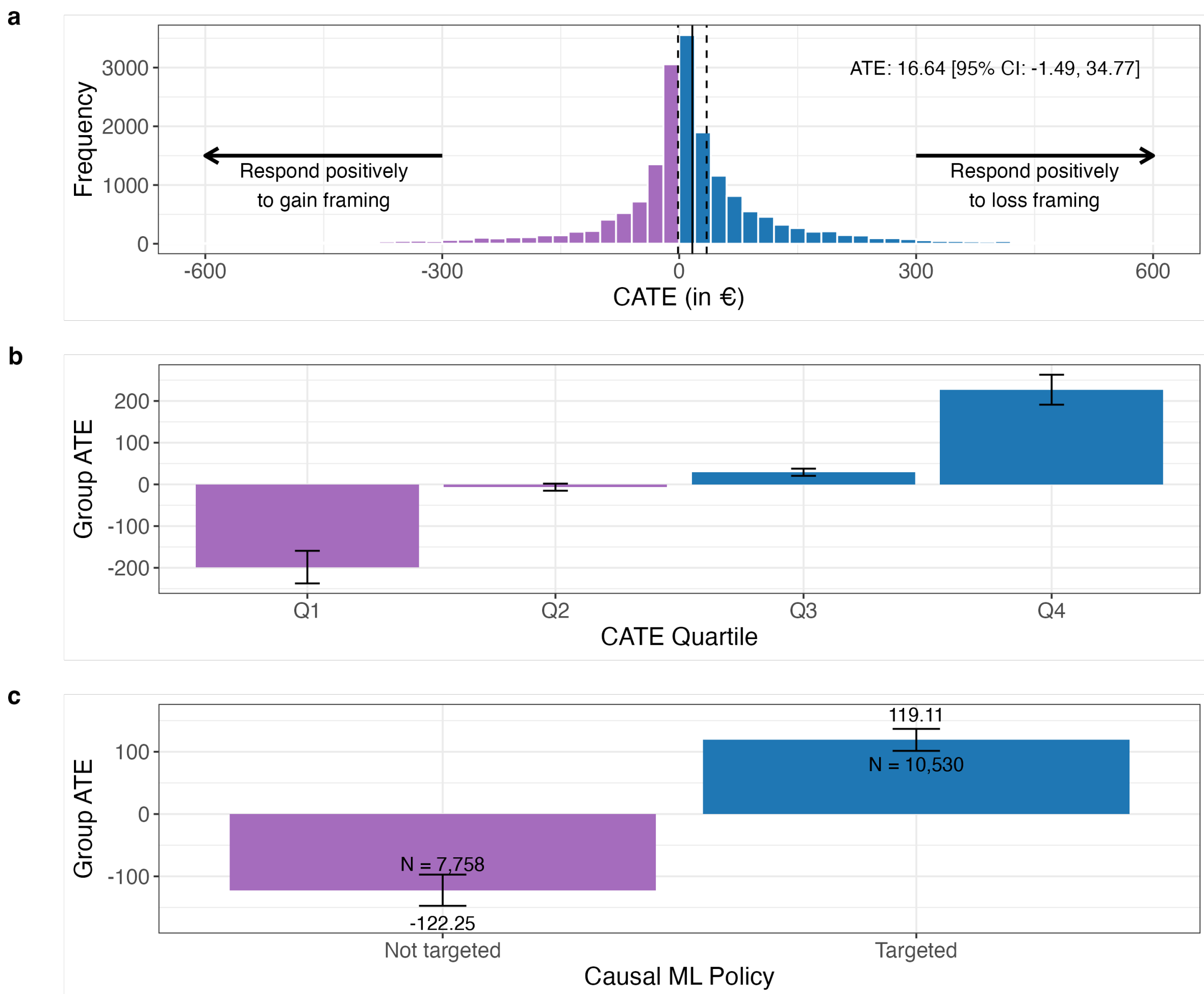

Figure S19: **Heterogeneous effects of loss versus gain framing on savings amount (intensive margin) unconditional on conversion.** This figure shows heterogeneity in the estimated effect of message framing on customers' retirement savings amounts among all customers, that is, converters and non-converters. The estimates are based on a causal forest that was trained on data from Field Experiment 1 and evaluated out-of-sample using data from Field Experiment 2. **a:** Distribution of CATEs of sending a loss-framed message (versus a gain-framed message) on savings amount. The x-axis is clipped at €600 for readability and omits outliers. **b:** ATE of sending a loss-framed message on the savings amount aggregated by CATE quartiles. **c:** Evaluation of a Causal ML–based targeting policy. The uncertainty margins depicted in the plots represent 95% confidence intervals.

Parametric benchmark analyses

Following Baiardi and Naghi [50], we benchmark the causal forest against fully saturated parametric models in which the treatment indicator is interacted with all 13 covariates. Specifically, a probit model for conversion and an OLS model for the saving amount among converters, both estimated on the data from Field Experiment 1 (see Table S7). A joint test of the 13 interaction terms does not reject homogeneous treatment effects on either margin ($\chi^2(13) = 10.39$, $p = 0.662$ for conversion; $F(13, 1167) = 1.19$, $p = 0.284$ for saving amount), and no individual interaction remains significant after Holm correction.

Table S7: **Saturated interaction models (Experiment 1) with treatment–covariate interactions.**

	Converted, Probit		Saving Amount (log), OLS	
Interaction with Loss	Coef. (SE)	p [Holm]	Coef. (SE)	p [Holm]
Gender (female)	−0.059 (0.072)	0.412 [1.000]	0.093 (0.191)	0.624 [1.000]
Age	0.002 (0.004)	0.541 [1.000]	0.000 (0.009)	0.969 [1.000]
Employment Status: Employed	0.080 (0.121)	0.508 [1.000]	0.448 (0.382)	0.241 [1.000]
Contract Valuation	0.039 (0.586)	0.947 [1.000]	−1.458 (1.509)	0.334 [1.000]
Number of Historical Payments	0.035 (0.046)	0.448 [1.000]	−0.009 (0.070)	0.902 [1.000]
Handling and Moving Skills	−0.241 (0.320)	0.450 [1.000]	−1.822* (0.944)	0.054 [0.700]
Information Skills	0.603* (0.326)	0.064 [0.829]	−0.796 (0.845)	0.347 [1.000]
Computer Skills	−0.231 (0.530)	0.663 [1.000]	1.875 (1.329)	0.158 [1.000]
Construction Skills	−0.645 (0.556)	0.246 [1.000]	3.112* (1.684)	0.065 [0.778]
Management Skills	−0.403 (0.359)	0.262 [1.000]	−0.880 (0.932)	0.345 [1.000]
Machinery and Equipment Skills	−0.069 (0.470)	0.883 [1.000]	1.149 (1.340)	0.391 [1.000]
Assisting and Caring Skills	0.510* (0.308)	0.098 [1.000]	−0.498 (0.857)	0.561 [1.000]
Communic., Collab., and Creativity Skills	0.036 (0.247)	0.884 [1.000]	−0.034 (0.649)	0.958 [1.000]
Joint test of interactions	$\chi^2(13) = 10.39$, $p = 0.662$		$F(13, 1167) = 1.19$, $p = 0.284$	
Observations	22,919		1,195	

Fully saturated models on Field Experiment 1 in which the loss-framing indicator is interacted with all 13 covariates used in the causal forest (covariate main effects included but omitted from the display). Unadjusted p-values with Holm-adjusted values in brackets. Statistical significance markers: * 0.10, ** 0.05, *** 0.01.

We then use the fitted models to predict individual-level treatment effects for every customer in Field Experiment 2, computed as the difference between the predicted outcomes under loss and under gain framing (for the saving amount, from a saturated OLS in euro levels so that predictions are on the same scale as the forest CATEs). To assess the economic impact of a policy based on these models, the predictions from the parametric models are evaluated with the same procedures as the causal forest CATEs. The two rankings correlate only moderately (Spearman rank correlation of 0.12 for conversion and 0.43 for the saving amount). The estimated treatment effects increase across quartiles of the parametric predictions, but in contrast to the causal forest the associated RATE is not significantly different from zero on either margin (conversion: 0.76, 95% CI: −0.23, 1.74; saving amount: €285, 95% CI: −€42, €612). In the off-policy evaluation, constructed identically to Tables 1 and 2, a targeting policy based on the parametric predictions yields 867 expected conversions and €1,785,426 in total savings—above uniform loss framing (829 conversions, €1,631,150) but well below the causal forest policy (1,255 conversions, €1,931,571).
